# The metabolome as a link in the genotype-phenotype map for peroxide resistance in the fruit fly, *Drosophila melanogaster*

**DOI:** 10.1186/s12864-020-6739-1

**Published:** 2020-05-04

**Authors:** Benjamin R. Harrison, Lu Wang, Erika Gajda, Elise V. Hoffman, Brian Y. Chung, Scott D. Pletcher, Daniel Raftery, Daniel E. L. Promislow

**Affiliations:** 1 Department of Pathology, University of Washington School of Medicine, Seattle, WA 98195 USA; 2 Department of Environmental and Occupational Health Sciences, University of Washington, Seattle, WA 98105 USA; 3 Department of Molecular and Integrative Physiology, University of Michigan, Ann Arbor, MI 48109 USA; 4 Northwest Metabolomics Research Center, Department of Anesthesiology & Pain Medicine, University of Washington, Seattle, WA 98195 USA; 5 Department of Biology, University of Washington, Seattle, WA 98195 USA

**Keywords:** Genetic variation, Complex trait, Endophenotype, Metabolome, GWAS, Quantitative genetics, Drosophila, Hydrogen peroxide, Oxidative stress, Starvation

## Abstract

**Background:**

Genetic association studies that seek to explain the inheritance of complex traits typically fail to explain a majority of the heritability of the trait under study. Thus, we are left with a gap in the map from genotype to phenotype. Several approaches have been used to fill this gap, including those that attempt to map endophenotype such as the transcriptome, proteome or metabolome, that underlie complex traits. Here we used metabolomics to explore the nature of genetic variation for hydrogen peroxide (H_2_O_2_) resistance in the sequenced inbred *Drosophila* Genetic Reference Panel (DGRP).

**Results:**

We first studied genetic variation for H_2_O_2_ resistance in 179 DGRP lines and along with identifying the insulin signaling modulator *u-shaped* and several regulators of feeding behavior, we estimate that a substantial amount of phenotypic variation can be explained by a polygenic model of genetic variation. We then profiled a portion of the aqueous metabolome in subsets of eight ‘high resistance’ lines and eight ‘low resistance’ lines. We used these lines to represent collections of genotypes that were either resistant or sensitive to the stressor, effectively modeling a discrete trait. Across the range of genotypes in both populations, flies exhibited surprising consistency in their metabolomic signature of resistance. Importantly, the resistance phenotype of these flies was more easily distinguished by their metabolome profiles than by their genotypes. Furthermore, we found a metabolic response to H_2_O_2_ in sensitive, but not in resistant genotypes. Metabolomic data further implicated at least two pathways, glycogen and folate metabolism, as determinants of sensitivity to H_2_O_2_. We also discovered a confounding effect of feeding behavior on assays involving supplemented food.

**Conclusions:**

This work suggests that the metabolome can be a point of convergence for genetic variation influencing complex traits, and can efficiently elucidate mechanisms underlying trait variation.

## Background

Phenotypic variation among individuals in a population arises from variation in the genotype, the environment, and the interaction between the two. Genetic variation is a major determinant of many complex traits and, while numerous genetic association studies have failed to explain substantial portions of heritable variation in a given complex trait, use of highly polygenic models have closed this gap considerably [1]. However, highly polygenic models do not easily allow us to identify gene-level associations with traits, and may not yield mechanistic insight into the pathways that shape trait variation. Genetic variation ultimately affects phenotype though the effect of genes on downstream ‘endophenotypes’ — the epigenome, transcriptome, proteome, metabolome and microbiome [2, 3]. Several authors have proposed that these endophenotypes, and the metabolome in particular, may serve as a powerful tool in mapping genotype to phenotype, as well as a source of information upon which to construct mechanistic hypotheses [3–7]. In this study, we explore the possibility that genetic effects on phenotypes are filtered through the profile of small molecules, the metabolome, that function downstream of genotype but upstream of phenotype.

The metabolome consists of the small biomolecules, typically less than 1500 Da, that make up the energetic, structural and functional building blocks of all life [8–12]. Given the role of these molecules in cells, researchers have pointed to the metabolome as a key link between genotype and phenotype [3–5, 13–15]. Genetic variation clearly influences the metabolome. For example, genome-wide association studies (GWAS) have identified alleles that potentially explain up to 60 to 80% of the variance in individual features in the human or plant metabolome [16–20]. Some have proposed mapping variation in the abundance of trait-associated metabolites in order to map genotype-to-metabolite-to-phenotype [2, 3, 5, 15, 21].

To explore the potential of metabolomic profiling to bridge the genotype-phenotypic gap and to identify underlying mechanisms of natural variation, here we study resistance to peroxide (H_2_O_2_) stress in a fruit fly model of genetic variation, the *Drosophila* Genetic Reference Panel (DGRP) [22]. This system provides an ideal model to study the ability of metabolic profiling to bridge the genotype-phenotype gap. First, H_2_O_2_ resistance assays can be performed on hundreds of flies in parallel, and the resulting survival data can be analyzed within a rigorous statistical framework [23–25]. Second, many association studies have examined genetic variation for survival in *Drosophila* [26–28], including in response to oxidative stressors [25, 29]. The DGRP, a set of genetically diverse and fully sequenced inbred lines, now enables labs around the world to quickly identify loci associated with any trait of interest [26, 30]. Third, numerous studies over the past decade have shown that metabolite profiles in flies are highly sensitive to variation due to genotype and environment [9, 31–36]. And so, a metabolomic study of H_2_O_2_ resistance in the DGRP could be used to simultaneously measure the metabolomic response to stress and its association with resistance phenotype within a genetically diverse population. We hypothesize that resistance to H_2_O_2_ among diverse genotypes may associate with metabolic pathways that mediate the resistance phenotype. This hypothesis supposes that we would find resistance-associated metabolic pathways among a panel of genetically diverse lines. Alternatively, metabolites could have genotype-specific associations to an extent that we may fail to detect trait associations with metabolic pathways across the genotypes measured here. We note that our experimental design does not rule out genotype-specific metabolic activity as a mediator of stress resistance. Rather, we seek to investigate the potential for trait-associated genotypes to converge on the metabolome.

We present an analysis of survival time and metabolic profiles in flies from DGRP lines held on H_2_O_2_ or control food. We show that survival is heritable, and that 34.2% of the total variance could be explained by additive effects of the known genetic variants in our study population. By mapping genetic variation associated with resistance, we identified at least two genes associated with lifespan on H_2_O_2_ food, including *NPF* and *u-shaped (ush*). To simultaneously assess the contribution of the metabolome to phenotypic variation, we profiled the metabolome of a subset of genotypes. By comparing highly resistant with highly sensitive lines, we modeled H_2_O_2_ resistance as a binary trait. Consistent with the hypothesis that genotypes could converge on common metabolomic endophenotypes, we found a consistent metabolomic signature of resistance to H_2_O_2_. Multivariate analysis of metabolome variation across these genotypes allows us to distinguish resistant from sensitive lines, even in samples of flies not exposed to H_2_O_2_ food. We compare the potential of genotype and metabolome to explain trait variation among the resistant and sensitive lines. Whereas multivariate clustering based on the metabolome leads to clear distinctions between resistant and sensitive genotypes, similar methods applied to genetic variation alone fail to differentiate their resistance phenotype. These results suggest that a variety of even quite diverse genotypes that share a phenotype may do so by converging on a similar metabolomic endophenotype.

Additionally, using univariate analysis of individual metabolite features, we found glycogen and folate metabolism are associated with stress resistance and validate this analysis by showing that metabolite feeding or genetic manipulation of candidate pathways both affect survival on H_2_O_2_ food.

Finally, we found a strong effect of H_2_O_2_ on feeding behavior, suggesting that variation in survival of flies on food supplemented with H_2_O_2_ could be explained in part by variation in starvation resistance. Our results suggest that starvation or nutrient assimilation might be the underlying cause of mortality in historical assays where the stressor has been administered to *Drosophila* in the food.

## Results

### Resistance to peroxide food within the DGRP

We found substantial variation among 179 DGRP lines for survival of adult females on H_2_O_2_ food. We used a mixed model to study the variation in mean lifespan and, in addition to significant effects of genotype (random effect, likelihood ratio *χ*^*2*^ = 36.2, df = 1, *P* = 1.7 × 10^− 9^, Methods), we found that weight was a significant predictor, with larger flies surviving longer on H_2_O_2_ (fixed effect, *β* = 0.49, *P* = 1.7 × 10^− 4^). We used individual fly lifespans to estimate a broad sense heritability for survival on H_2_O_2_ food of *H*^*2*^ = 44.8% ± 0.04 (mean ± se, Methods).

**Fig. 1:**
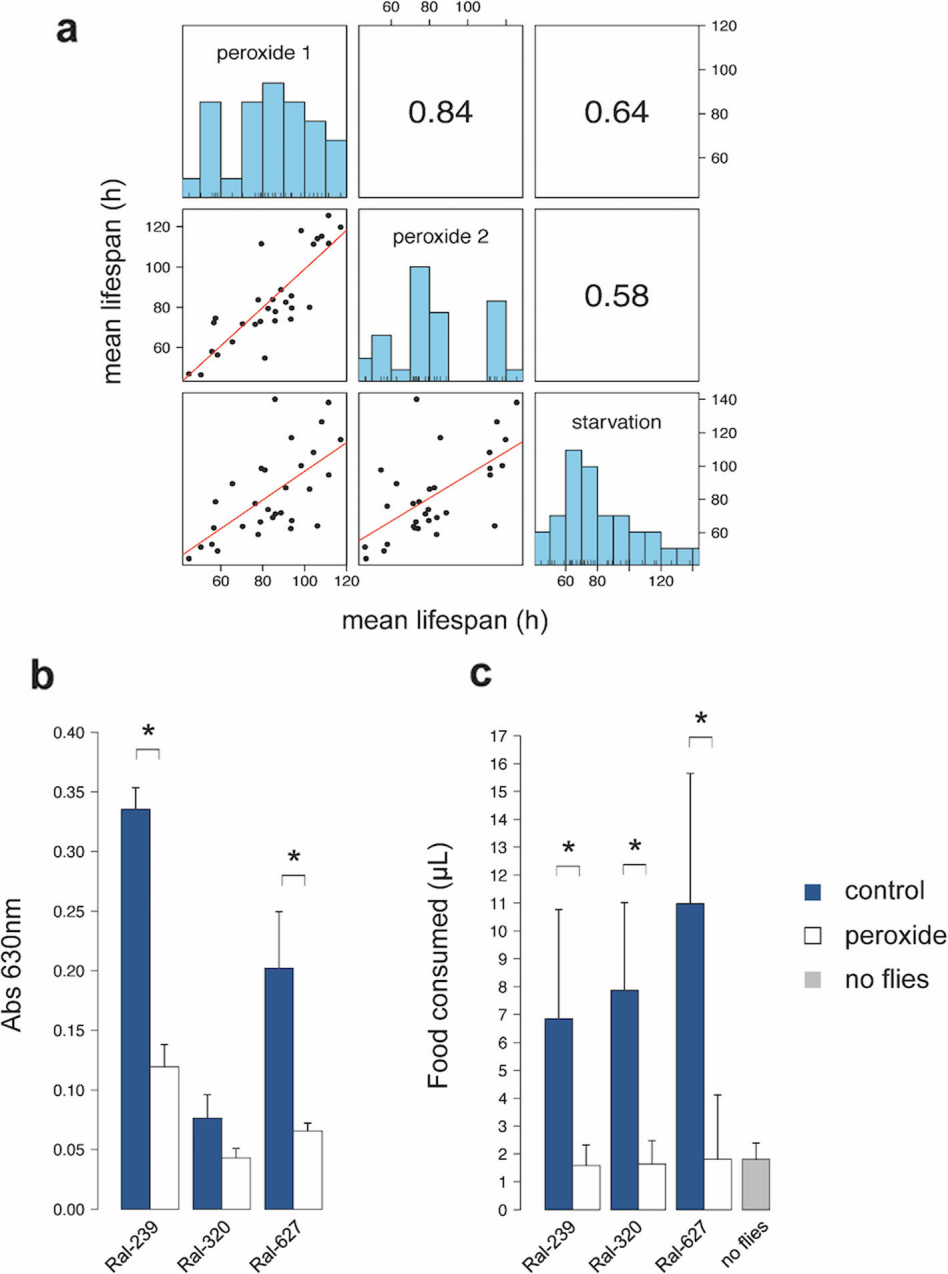
Starvation explains the lifespan effect of H_2_O_2_ food (**a**) The correlations between mean lifespan of 31 DGRP lines on food containing either 2% glucose and 2% hydrogen peroxide in replicate trials (peroxide 1 and peroxide 2) or no glucose (starvation). Below the diagonal are plots of trait values (mean survival (h)). Least-squares linear regression lines are shown in red. Above the diagonal are Spearman correlation coefficients for each pair of traits. **b** and **c** Feeding assays, the mean (+SD) absorbance at 630 nm of extracts from three replicate vials of seven to eleven 1 to 5 day old mated females from the indicated DGRP line (**b**). Flies were exposed to peroxide (open bars) or control food (colored bars) for two hours of feeding prior to dye extraction. **c** The mean (+SD) volume of liquid food consumed by ten replicates of ten 2 to 4 day-old mated females was measured using the CAFE assay. CAFE food contained 2% glucose and, either water (colored bars) or 2% peroxide (open bars). Additional apparatus were set up without flies (no flies) to measure volume loss due to evaporation. Asterisks indicate p < 0.05 (Welch’s t-test)

We then compared our measures of H_2_O_2_ resistance in the DGRP with traits measured in the DGRP in previous studies. These studies have measured either survival or behavioral responses of the DGRP to two other oxidative stressors, paraquat and menadione [25, 29]. We observed Pearson correlations of *r* = 0.34 (*n* = 179, *P* < 10^− 4^) and *r* = 0.35 (n = 179, *P* < 10^− 4^) between the mean survival time reported here and those measured on food supplemented with paraquat or menadione, respectively (Fig. S1 [25]). We found no correlation between our measures of H_2_O_2_ survival and two behavioral traits, the startle response and climbing, measured by Jordan et al. [29] following chronic (13–16 day) exposure to menadione food (data not shown). We also found that the survival times of the DGRP on H_2_O_2_ food correlate highly with survival measured under starvation in two different labs (Fig. S1 [26, 37]) as well as data from our own lab (Fig. 1a). It is notable that the correlation between H_2_O_2_ resistance and starvation was greater than the correlation between H_2_O_2_ resistance and any other trait published for the DGRP, including survival on food containing paraquat or menadione (Fig. S1). These results suggest that H_2_O_2_ food may affect feeding or nutrient assimilation in *Drosophila*.

To test the possibility that H_2_O_2_ affects feeding behavior, we measured the amount of food consumed by flies in the CAFE and dye incorporation assays described in the Methods section [38, 39]. Peroxide reduced feeding in each of three different genotypes tested, including strains with both relatively long and short survival time on H_2_O_2_ food (Fig. 1b and c). During the 24 h feeding period in the CAFE assay, flies exposed to liquid H_2_O_2_ food consumed no more than the volume lost due to evaporation in chambers without flies, suggesting that the flies consumed very little H_2_O_2_ food over 24 h (Fig. 1c). This finding suggests that mortality in flies exposed to H_2_O_2_ is due at least in part to starvation, in addition to any oxidative stress caused by H_2_O_2_ exposure.

### Genetic associations with peroxide resistance

The DGRP is both highly inbred and sequenced to high confidence for the majority of SNPs and small indels [22], therefore we sought to estimate the extent of genetic variation captured by the characterized genetic variation in the DGRP. We used restricted maximum likelihood to estimate the proportion of variance in phenotype that could be explained by the genomic relationship between the lines used in our study, the so-called SNP heritability (${\hat{h}}_{SNP}^2$) at 34.2% (Methods). Thus, a substantial amount of the heritable variation in H_2_O_2_ resistance could potentially be explained by the characterized genetic variation in the DGRP. We then sought to identify individual genetic variants and genes that might associate with H_2_O_2_ resistance.

**Fig. 2:**
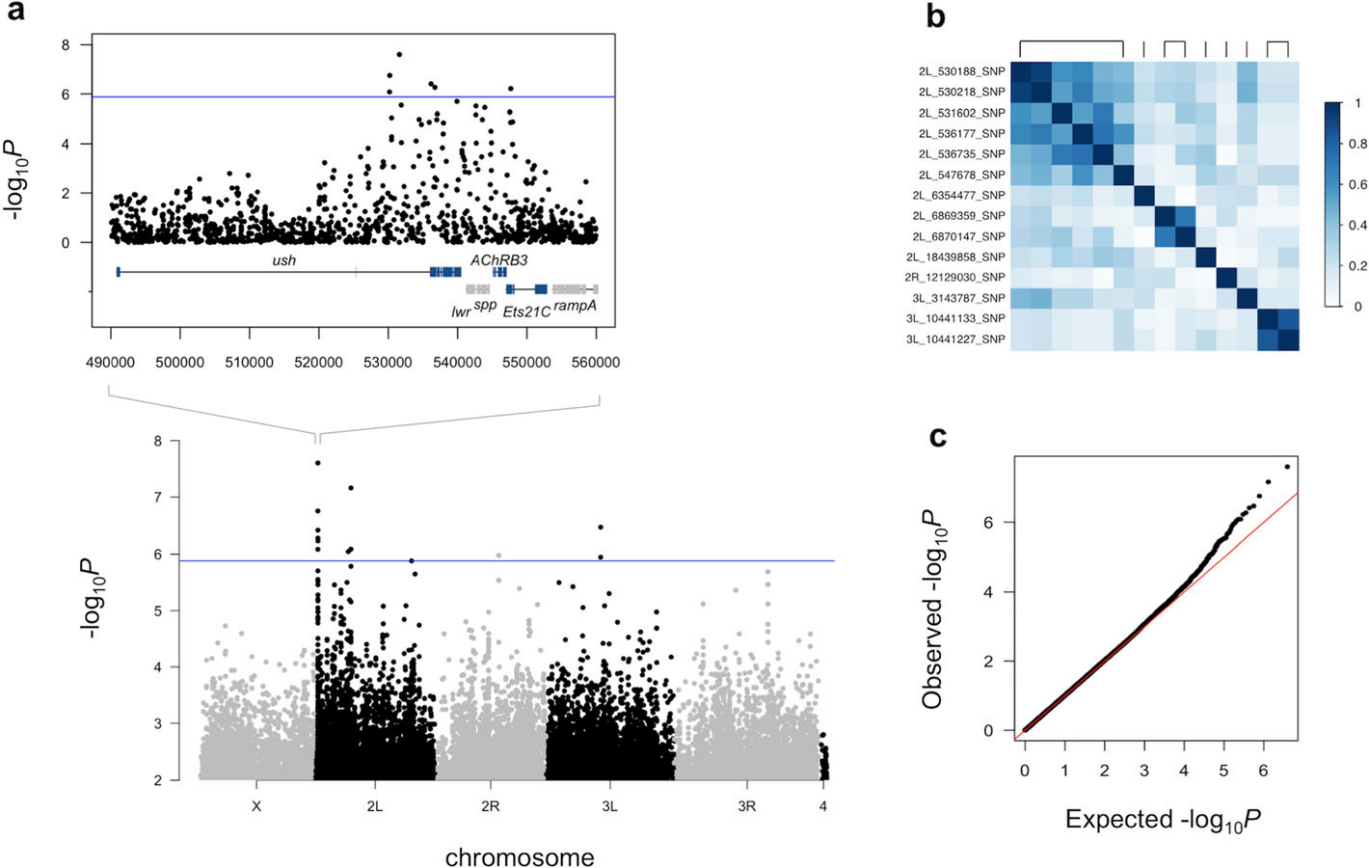
Genome-Wide Association (**a**) –log_10_ *P*-values for each polymorphism (MAF ≥ 0.05, < 30% missing) in association with lifespan on H_2_O_2_ food, *P*-values above 0.01 not shown, the FDR of 0.2 level of significance is shown by a blue line. Inset shows a locus on chromosome 2 L that includes 6 resistance-associated SNPs in LD (*r* ≥ 0.5) and several candidate genes, including *ush*. Genes shown in blue were significant in gene-level analysis (FDR < 0.5). **b** A pairwise LD plot for all 14 resistance-associated SNPs (FDR < 0.2), groups of SNPs in LD are indicated with bars across the top, and a color scale for *r* is shown. **c** A Q-Q plot of –log_10_ P-values for the variants tested in the GWAS showing little to no inflation after adjusting for population structure (Methods)

We used a linear regression model in PLINK [40] to test for associations of H_2_O_2_ resistance with approximately 1.9 million SNPs with a MAF ≥ 5% and <30% missing genotypes, while accounting for population structure and a significant effect of the major inversion *In(2 L)t* (Methods). We used the *q* value approach [41] to control the false discovery rate (FDR) and at 20% FDR, 14 variants (all were SNPs) were associated with resistance (Fig. 2a, Table S1). Pairwise linkage disequilibrium (LD) among the 14 SNPs indicates that they associate with H_2_O_2_ resistance as seven loci, or groups of SNPs in LD (*r*^*2*^ > 0.5, Fig. 2b). With only 179 genotypes, we lack the power to analyze SNP-SNP interactions among these loci. However, our data indicate that H_2_O_2_ resistance is polygenic within the DGRP.

To investigate these genetic associations further, we performed gene ontology (GO) analysis, looking for biological processes and signaling pathways that are over-represented among the markers associated with survival on H_2_O_2_ food. To identify gene-level associations, many studies use the minimum *P*-value of all variants in a gene (*P*_*min*_). One might expect bias in this approach, such that genes with more variants are more likely to have a smaller (more significant) *P*_*min*_ by chance alone. Indeed, we found that -log_10_(*P*_*min*_) was positively associated with the number of variants per gene (Fig. S2), potentially biasing gene-trait associations in favor of genes with more variants [42]. To test for this bias, we compared the top 200 genes ranked by *P*_*min*_ with the top 200 genes from ten GWAS of randomly permuted phenotypes. Out of 15,322 gene models in the Fly Base release 5.49, the null expectation for such intersection would be 2.6 genes. In contrast, we found an average of 11.6 ± 2.0 genes (mean ± se) in common across the permutations (*χ* ^*2*^ = 28.3, df = 3, *P* < 1.1 × 10^− 7^), consistent with a bias caused by SNP density. To correct for this bias, we used a permutation approach to derive gene-level *P*-values, *P*_*gene*_, while also accounting for population structure (Methods). Unlike *P*_*min*_, *P*_*gene*_ did not associate with the number of variants per gene, and it reduced the number of false-positives when compared to top genes from GWAS of randomized phenotypes (Fig. S2, *χ* ^*2*^ = 2.2 × 10^− 26^, df = 3, *P* = 1). Thus, *P*_*gene*_ increases the accuracy of gene-trait associations. Only one gene, *ppk14*, was significant below an FDR of 0.05, so we chose to look for biological processes or pathways that might be enriched among the 29 genes at FDR ≤ 0.5 (Table 1). This gene ontology (GO) enrichment analysis identified several biological processes and two biological pathways (FDR ≤ 0.05, Table 2). Most of the enriched processes are nested in hierarchical categories and thus are not independent. Also, the enrichment of 7 of the 9 biological processes, and the endothelin signaling pathway, is due entirely to three genes occurring in a small gene cluster, each encoding adenylyl cyclase (ACXA, ACXB and ACXE, Table 2). Adenylyl cyclase is involved in several signaling pathways, including G-protein coupled and calcium-based signaling. Separately, the platelet-derived growth factor (PDGF) signaling pathway is enriched (FDR = 0.02) due to three other genes in our dataset, *Rab2*, *Ets21C* and the c-Myc-binding protein homolog CG17202.

**Table 1:** Genes Associated with Peroxide Resistance

FlyBase ID	*Gene*	Name	Lead Variant	*P*_*min*_	*P*_*gene*_	FDR
FBgn0031803	*ppk14*	Pickpocket 14	2L_6353972_SNP	2.12 × 10–6	9.00 × 10–6	0.004797
FBgn0031261	*nAChRbeta3*	Nicotinic acetylcholine receptor beta 3 (Dbeta3) subunit	2L_544231_SNP	9.73 × 10–7	2.20 × 10–5	0.011704
FBgn0031802	*ppk7*	Pickpocket 7	2L_6351358_SNP	2.12 × 10–6	2.70 × 10–5	0.014337
FBgn0034098	*krimp*	FI20010p1	2R_12124705_SNP	1.68 × 10–6	3.10 × 10–5	0.01643
FBgn0034099	*CG15708*	AT21920p	2R_12127442_SNP	1.68 × 10–6	3.10 × 10–5	0.01643
FBgn0005660	*Ets21C*	DNA-binding protein D-ETS-6	2L_546065_SNP	9.73 × 10–7	3.30 × 10–5	0.017424
FBgn0002031	*l(2)37Cc*	Protein l(2)37Cc	2L_19121067_SNP	7.57 × 10–6	4.50 × 10–5	0.023715
FBgn0003963	*ush*	Zinc finger protein ush	2L_475238_SNP	4.51 × 10–8	7.10 × 10–5	0.037346
FBgn0031865	*Nha1*	Na[+]/H[+] hydrogen antiporter 1, isoform A	2L_6860010_SNP	5.32 × 10–8	0.000123	0.064575
FBgn0020906	*Jon25Bi*	Jonah 25Bi	2L_4953449_SNP	4.23 × 10–5	0.000214	0.112136
FBgn0031655	*Marcal1*	SWI/SNF-related matrix-associated actin-dependent regulator of chromatin subfamily A-like protein 1	2L_4954910_SNP	1.27 × 10–5	0.000253	0.132319
FBgn0263831	*Gen*	Flap endonuclease GEN	3L_5141974_SNP	7.43 × 10–6	0.000262	0.136764
FBgn0037730	*DmelCG9444*	CG9444, isoform A	3R_5506170_SNP	9.71 × 10–5	0.000272	0.141712
FBgn0005616	*msl-2*	E3 ubiquitin-protein ligase msl-2	2L_3461213_SNP	3.65 × 10–5	0.000367	0.19084
FBgn0263102	*psq*	Pipsqueak, isoform M	2R_6444567_SNP	3.29 × 10–5	0.000373	0.193587
FBgn0004103	*Pp1-87B*	Serine/threonine-protein phosphatase alpha-2 isoform	3R_8248858_SNP	0.0001966	0.000437	0.226366
FBgn0083960	*DmelCG34124*	FI23230p1	2L_4957415_SNP	1.27 × 10–5	0.000461	0.238337
FBgn0040510	*ACXA*	Adenylyl cyclase X, isoform A	2L_12920145_SNP	5.17 × 10–5	0.000472	0.243552
FBgn0085227	*CG34198*	HDC07368	2R_15569059_SNP	0.0001068	0.000489	0.251835
FBgn0040506	*ACXE*	Adenylyl cyclase X, isoform E	2L_12924675_SNP	8.53 × 10–5	0.000517	0.265738
FBgn0027109	*NPF*	Neuropeptide F	3R_12434359_SNP	9.19 × 10–5	0.000578	0.296514
FBgn0031538	*DmelCG3246*	CG3246	2L_3459329_SNP	3.65 × 10–5	0.000611	0.312832
FBgn0052391	*DmelCG32391*	Uncharacterized protein	3L_7060319_SNP	1.15 × 10–5	0.000723	0.369453
FBgn0038043	*CG17202*	c-Myc-binding protein homolog	3R_8247496_SNP	0.0001966	0.000762	0.38862
FBgn0005640	*Eip63E*	Ecdysone-induced protein 63E, isoform N	3L_3514849_SNP	1.80 × 10–5	0.00091	0.46319
FBgn0051641	*stai*	stathmin	2L_6099379_SNP	6.01 × 10–6	0.000922	0.468376
FBgn0014009	*Rab2*	GH01619p	2R_2583312_SNP	5.43 × 10–5	0.000932	0.472524
FBgn0262467	*Scox*	AT19154p	2L_4967292_SNP	0.0002591	0.000943	0.477158
FBgn0040509	*ACXB*	Adenylyl cyclase X, isoform B	2L_12916421_SNP	0.0003891	0.00096	0.4848

The gene identifications from Fly Base (FB) release 5.49 for all genes with *P*_*gene*_ FDR < 0.5. The variant with the smallest *P* value for each gene (*P*_*min*_) is listed as the lead variant

**Table 2:** Process and Pathway Enrichment Among Candidate Genes

PANTHER GO-Slim Biological Process	Gene Content	Input	Expected	Fold Enrichment	*P*-value	FDR
activation of adenylate cyclase activity (GO:0007190)	32	3*	0.07	46.09	4.69 × 10 ^− 5^	0.0353
adenylate cyclase-activating G-protein coupled receptor signaling pathway (GO:0007189)	32	3*	0.07	46.09	4.69 × 10 ^−5^	0.0235
cAMP-mediated signaling (GO:0019933)	41	3*	0.08	35.98	9.38 × 10 ^−5^	0.0282
regulation of cAMP-mediated signaling (GO:0043949)	41	3*	0.08	35.98	9.38 × 10 ^−5^	0.0235
regulation of adenylate cyclase activity (GO:0045761)	42	3*	0.09	35.12	1.00 × 10 ^−4^	0.0216
regulation of lyase activity (GO:0051339)	43	3*	0.09	34.3	1.07 × 10 ^−4^	0.0201
cyclic-nucleotide-mediated signaling (GO:0019935)	50	3*	0.1	29.5	1.64 × 10 ^−4^	0.0274
regulation of biological process (GO:0050789)	1359	11	2.76	3.98	3.87 × 10 ^−5^	0.0582
biological regulation (GO:0065007)	1492	11	3.03	3.62	9.15 × 10 ^−5^	0.0343
PANTHER Pathways	Gene Content	Input	Expected	Fold Enrichment	P-value	FDR
PDGF signaling pathway (P00047)	47	3	0.1	31.38	1.38 × 10^−4^	0.0213
Endothelin signaling pathway (P00019)	74	3*	0.15	19.93	0.0495	0.0256

Enrichment analysis for the 29 genes associated with peroxide resistance. Gene Content is the number of genes in the respective process/pathway in the *Drosophila* genome (*n* = 13,767 genes in the GO database)*.* Input is the number of resistance-associated genes in each process/pathway, and Expected is the number expected under the null hypothesis. Asterisks (*) indicate categories enriched due to the adenylyl cyclase X gene cluster

**Fig. 3:**
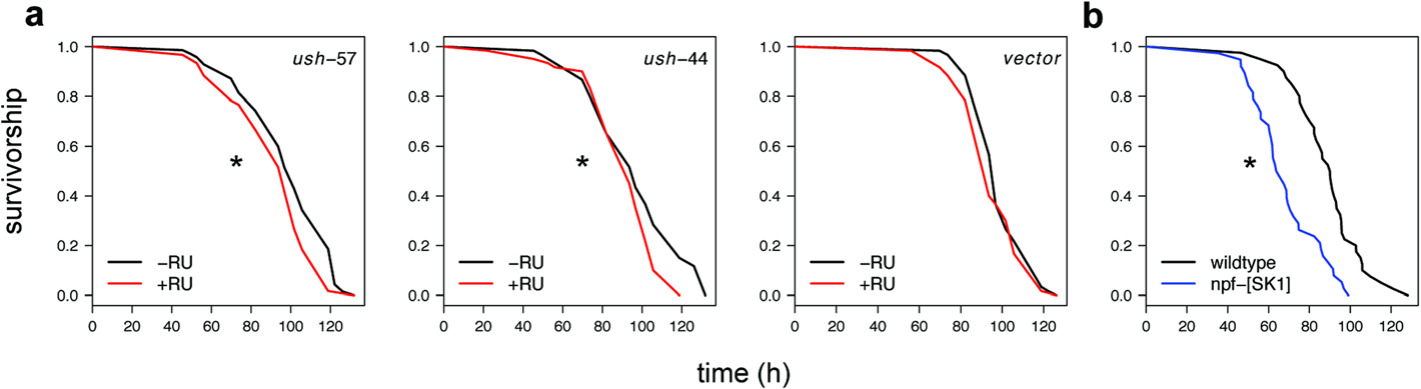
Candidate Gene Validation (**a**) Mated F_1_ females from crosses of the GeneSwitch106 driver to either UAS-RNAi lines targeting *u-shaped* (*ush-57* and *ush-44*) or a UAS-control line (attP2*, vector*), which were maintained on RU486 food (red line) or –RU food (black line) for 48 h prior to being transferred to H_2_O_2_ food or control food. Survivorship was recorded for six replicate vials of ten flies per vial on H_2_O_2_ food (*n* = 60 flies) and two replicate vials on control food (*n* = 20 flies). In another experiment (**b**), survivorship of wild type females (Canton-S, *n* = 40) was compared to *NPF*^*SK1*^ females (*n* = 38) on H_2_O_2_ food or control food in an activity monitoring system (Methods). Asterisks indicate significant effect of RU486, or of the *NPF*^*SK1*^ mutation (*P* < 0.05) from the log-rank test using the survival package in R. Results are representative of at least two independent experiments, and there was no mortality observed on control food during these experiments

We validated these gene-trait associations by using RNAi to manipulate the expression of six of the 29 candidate genes, *nAChRbeta3*, *Ets21C*, *ush*, *Nha1*, *Jon25Bi* and *Marcal1* (*P*_*gene*_ < 0.0003 in all cases), and testing the effect of a mutation in a seventh candidate *NPF* (*P*_*gene*_ < 0.0006). Several of the candidates reside near a cluster of six trait-associated SNPs within a 17.5 kb interval on chromosome 2 (Fig. 2a). This interval spans several genes, including *u-shaped (ush, P*_*gene*_ = 7.1 × 10^− 5^*)*, which contains an intronic C/T SNP associated with H_2_O_2_ resistance (*P* = 2.49 × 10^− 8^) and several other SNPs in LD with this SNP (Fig. 2b). None of these SNPs are predicted to alter amino acid sequence or splicing, but instead may affect *ush* expression. *Ush* has roles in development and growth, including as a negative regulator of PI3K activity within the IlS pathway in the fat body [43]. To validate the effects of *ush* on H_2_O_2_ resistance, we used the RU486-inducible GAL4 GeneSwitch driver S106 to drive RNAi targeting *ush* in the adult fat body [44]. RU486 treatment of flies carrying both S106 and UAS-RNAi targeting *ush* resulted in shorter survival times on H_2_O_2_ food than the same genotype without RU486 (Fig. 3). We saw the same result with two independent RNAi lines, each targeting a different portion of the *ush* mRNA (Fig. 3). We saw no effect of RU486 on the survival of F_1_ flies carrying the driver and the empty control P-element in the same genetic background as the UAS-RNAi flies, nor an effect of RNAi on lifespan on food lacking H_2_O_2_ (see Methods). Knocking down *ush* in the nervous system with the elav GeneSwitch driver did not affect H_2_O_2_ resistance (data not shown), and ubiquitous knockdown of *ush* appears to be lethal as we failed to recover Act-GAL4/*ush*-RNAi flies in crosses of the *ush*-RNAi construct to the constitutive Act-GAL4 driver. We also detected a strong interaction between RU486 treatment and an Act GeneSwitch driver in our H_2_O_2_ food assay and so were unable to test the effect of ubiquitous knockdown of *ush* in adult flies (data not shown). Similar RNAi of five other candidates did not affect H_2_O_2_ resistance (data not shown).

Another candidate, *NPF* (*P*_*gene*_ = 5.8 × 10^− 4^) encodes the ligand neuropeptide F (NPF), which controls feeding, ethanol sensitivity and other behaviors [45]. The *npf*^*SK1*^ deletion allele reduced H_2_O_2_ resistance when compared to wildtype control flies and did not affect survival on control food (Fig. 3 and data not shown). These data demonstrate that manipulation of candidate genes from our GWAS affects the H_2_O_2_ resistance phenotype.

### Metabolite profiles associated with peroxide resistance

To investigate the effect of H_2_O_2_ on the fly metabolome, and the potential for the metabolome to explain genetic variation in resistance to H_2_O_2_, we measured untargeted LC-MS profiles in three biological replicates for each of eight resistant (mean survival time = 106.9 h, range = 90.4–119.7 h) and eight sensitive (mean survival time = 58.9 h, range = 53.2–70.0 h) lines, chosen such that the two groups did not differ substantially in fly size (Fig. S3). These lines were subjected to another survival assay and, 24 h after being exposed to H_2_O_2_ or control food, samples of flies from each line and treatment were flash frozen for aqueous metabolite extraction, while survival measurements were conducted on the remaining flies.

**Fig. 4:**
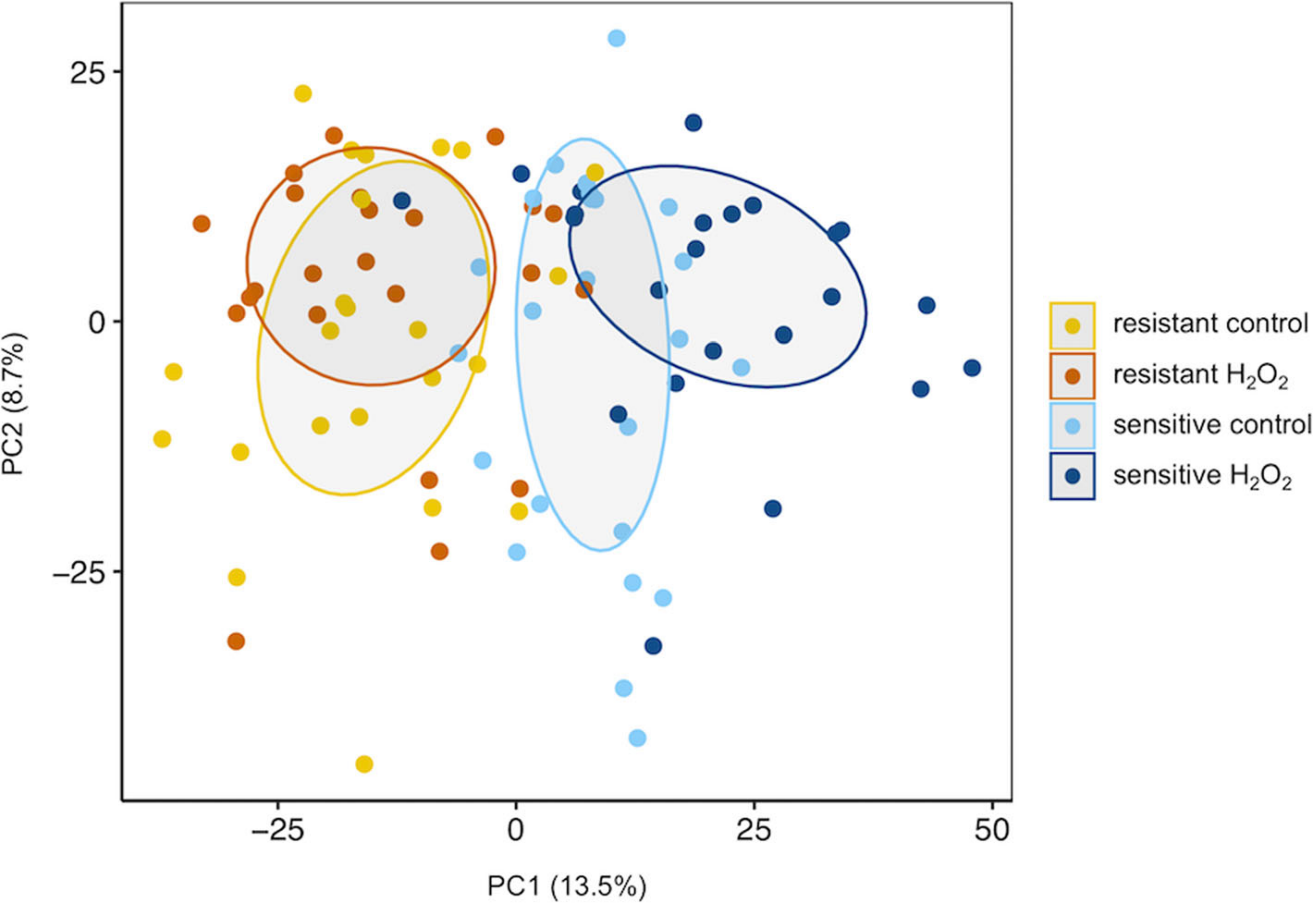
Projections of Metabolomic Principal Components The first and second principal components of the negative mode data labeled to indicate resistant and sensitive genotypes, as well as the effect of treatment (control vs. H_2_O_2_). Trait and treatment groups are indicated by colored points and ellipses (50% CI). The percentage of the variance explained by each PC is shown in parentheses

Global metabolite profiling measured 2722 and 2691 features that passed quality control thresholds from positive and negative ionization modes, respectively and were analyzed separately (Methods). We first explored these data using principal component analysis (PCA). Samples from resistant and sensitive genotypes clearly separate along principal component one of the negative mode (PC1_neg_, Fig. 4) and along PC2 of the positive mode (PC2_pos_, Fig. S4). Interestingly, H_2_O_2_-treatment samples appear distinct from untreated samples among the sensitive genotypes along both PC1_neg_ and PC2_pos_, and this separation is not apparent among the resistant genotypes (Fig. 4 and Fig. S4). Principal component analysis thus detected between-group variation in metabolite profiles from sensitive and resistant flies, and further suggested an effect of treatment on sensitive that is not seen in resistant flies.

**Fig. 5:**
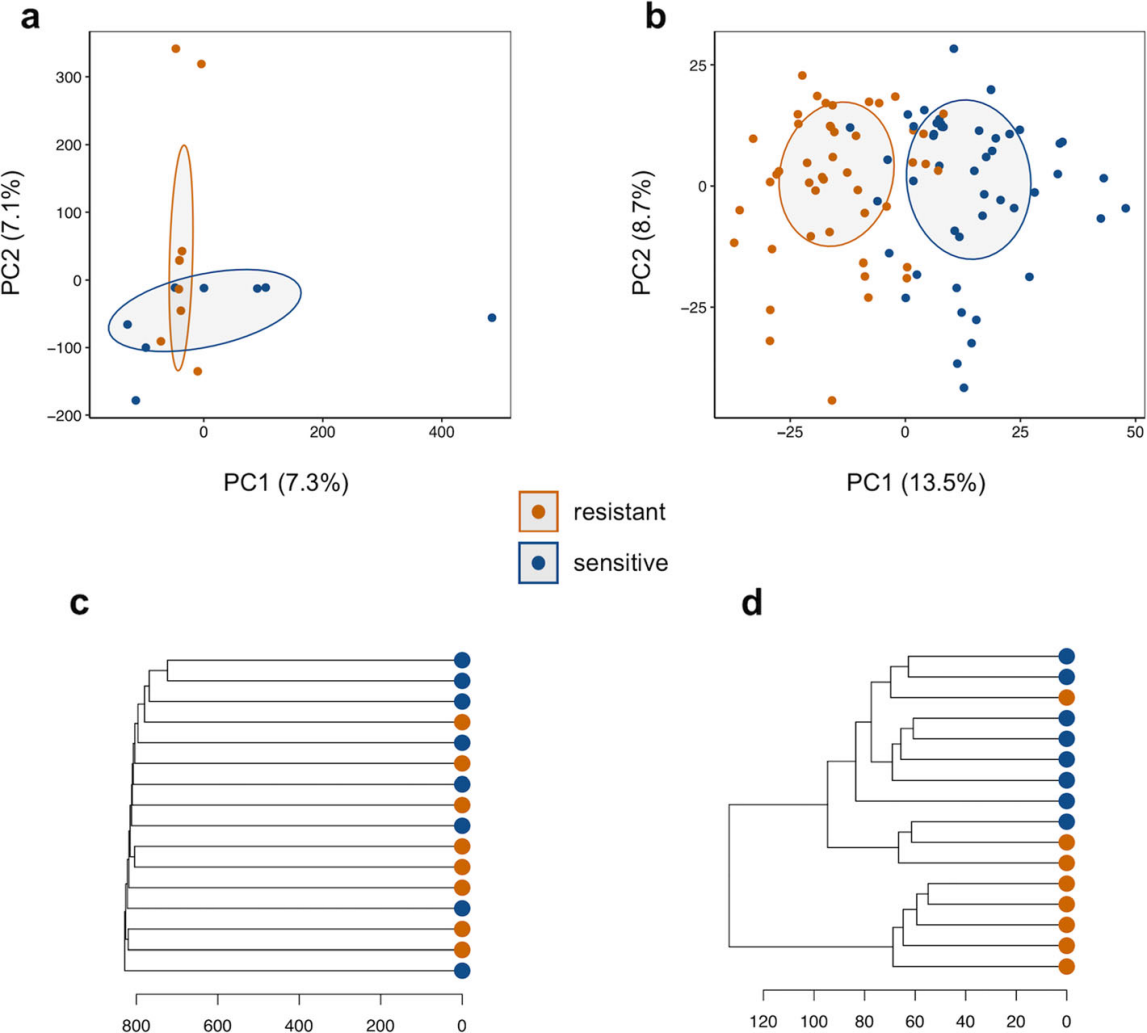
Metabolome is Proximal to Phenotype on the Genotype-Phenotype Map (**a**) PCs 1 and 2 of > 3.2 × 10^5^ LD-pruned variants (no missing calls, *r*^*2*^ < 0.5 within 50 kb windows) from the 16 genotypes in the metabolomics analysis. **b** PCs 1 and 2 of 2691 median normalized negative mode LC-MS features (corrected for batch and sample weight) from the same genotypes as in (**a**). In all plots, sensitive genotypes are colored in blue and resistant genotypes are colored in orange. The variance explained by each PC is shown in parentheses and the ellipses are the 50% confidence intervals. **c** Hierarchical clustering of the same genetic variants (**c**) or line mean values of the LC-MS features (**d**). Data were clustered using the minimal variance method (Ward’s D2) in the hclust R package

The separation of resistant and sensitive lines by metabolome profile is striking (Fig. 5b). However, the genotypes chosen for metabolite profiling were among the extremes of resistance to H_2_O_2_. This design raises the possibility that resistant and sensitive lines have a confounding genetic relationship. To determine if genotype could also separate lines chosen deliberately with extreme phenotypes, we carried out PCA and hierarchical clustering of the genotype data on the same lines. We analyzed the first ten PCs of genotype, which together account for 69% of the variance of > 3.2 × 10^5^ genetic variants in the 16 lines used for metabolomics. These PCs failed to clearly separate the resistant and sensitive flies (for example, PC1 vs. PC2 is shown in Fig. 5). Similarly, hierarchical clustering of these 16 lines using the same genetic variants also failed to separate the two phenotypes (Fig. 5). For comparison, clustering of 2691 negative mode metabolite features separated genotypes into two clusters composed of resistant and sensitive lines (Fig. 5). Thus, the distinction between resistant and sensitive flies is more obvious given an unbiased sample of LC-MS features rather than an unbiased sample of their genotype.

### Metabolic pathways associated with peroxide resistance

Principal component analysis suggested that the metabolome of sensitive and resistant lines differ systematically and moreover, that resistant and sensitive genotypes differ in their metabolic response to H_2_O_2_ treatment (Fig. 4). To identify the specific metabolites whose abundance was affected by treatment in a trait-dependent way we ran a linear regression model, predicting metabolite level in response to the interaction between the trait and treatment. In the negative mode data we found 105 features that were significant for a trait by treatment interaction (FDR < 0.1). Figure S4 shows the clustering of the features with a significant treatment by trait interaction term. These data come from analysis of profiles from negative mode only, as no features from positive mode reached our FDR cutoff for the interaction term. The clustering of features by z-score across samples revealed a consistent pattern with a substantially different effect of H_2_O_2_ on the metabolome of sensitive genotypes compared to resistant genotypes (Fig. S5). This pattern is similar to the apparent separation of samples across the latent variables revealed in PCA (Fig. 4).

We used the *mummichog* software package [46] to identify metabolomic pathways enriched among the features associated with H_2_O_2_ resistance, or among features with significant treatment by trait interaction effects. *Mummichog* matches m/z ratios and retention time data for the features to those predicted to occur if those features were enriched for a given metabolic pathway [46]. It is important to point out that we use *mummichog* as a tool to identify metabolic pathways that could associate with variation in H_2_O_2_ resistance and not as a tool for metabolite annotation. This analysis identified several pathways, including carbohydrate metabolism, amino acids and their biosynthesis, and folate metabolism (see Table S2, Additional File 2). Many of these pathways share overlapping metabolites and some pathways are nested within other pathways. We chose a subset of the identified pathways for further analysis based on a several criteria. One criterion was the significance of each pathway across the different contrasts in the linear model (see Table S2, Additional File 2). Another criterion was the strength of the identification; we gave higher priority to those features that were uniquely assigned to a particular metabolite or pathway, rather than features that were ambiguously associated with more than one metabolite, or with a metabolite that is present in several pathways.

### Glycogen metabolism

**Fig. 6:**
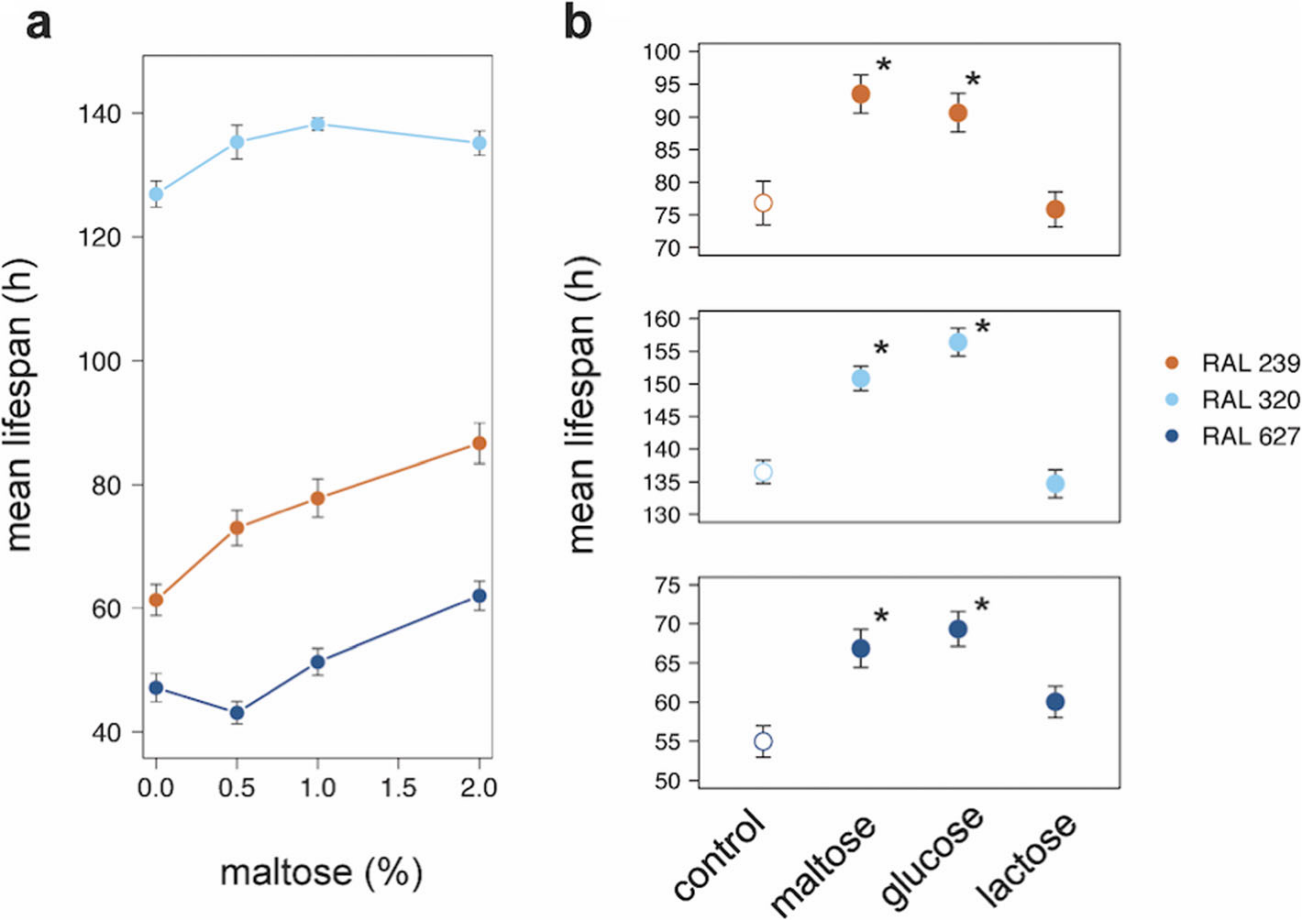
Supplemental Maltose or Glucose Increases Resistance to Peroxide. Females of three different DGRP lines were allowed to feed on media containing 2% glucose with the indicated amount of supplemental maltose (**a**). **b** Mated females fed on glucose food containing 2% of either additional maltose, additional glucose, or additional lactose, or control without additional carbohydrate for four days prior to being transferred to 2% glucose food containing 2% H_2_O_2_ for survival analysis*.* Points are the mean lifespans (± 1 s.e.) of 49 to 60 flies. ANOVA was used to compare the effect of maltose (*P* = 0.0035) after removing the effect of line in (**a**). The log-rank test was used in (**b**) to test the difference between lifespans of carbohydrate-fed verses control flies (asterisk, Bonferroni-corrected *P* ≤ 0.05)

Glycogen metabolism was identified by *mummichog* among the features that showed significant trait by treatment interaction (Fig. S6). Glycogen is a storage form source of glucose in *Drosophila* and variation in glycogen metabolism across the lines measured here may result in sensitivity to H_2_O_2_ food. Consistent with a role for glycogen in resistance to H_2_O_2_ food, flies fed supplemental maltose prior to being exposed to H_2_O_2_ showed increased resistance to H_2_O_2_ food in a dose-dependent manner (Fig. 6). Maltose could increase survival by providing glucose, or perhaps by some other effect as a disaccharide. Supplementing fly diet with glucose but not the disaccharide lactose extends survival time similar to supplemental maltose, which is consistent with the former hypothesis (Fig. 6).

**Fig. 7:**
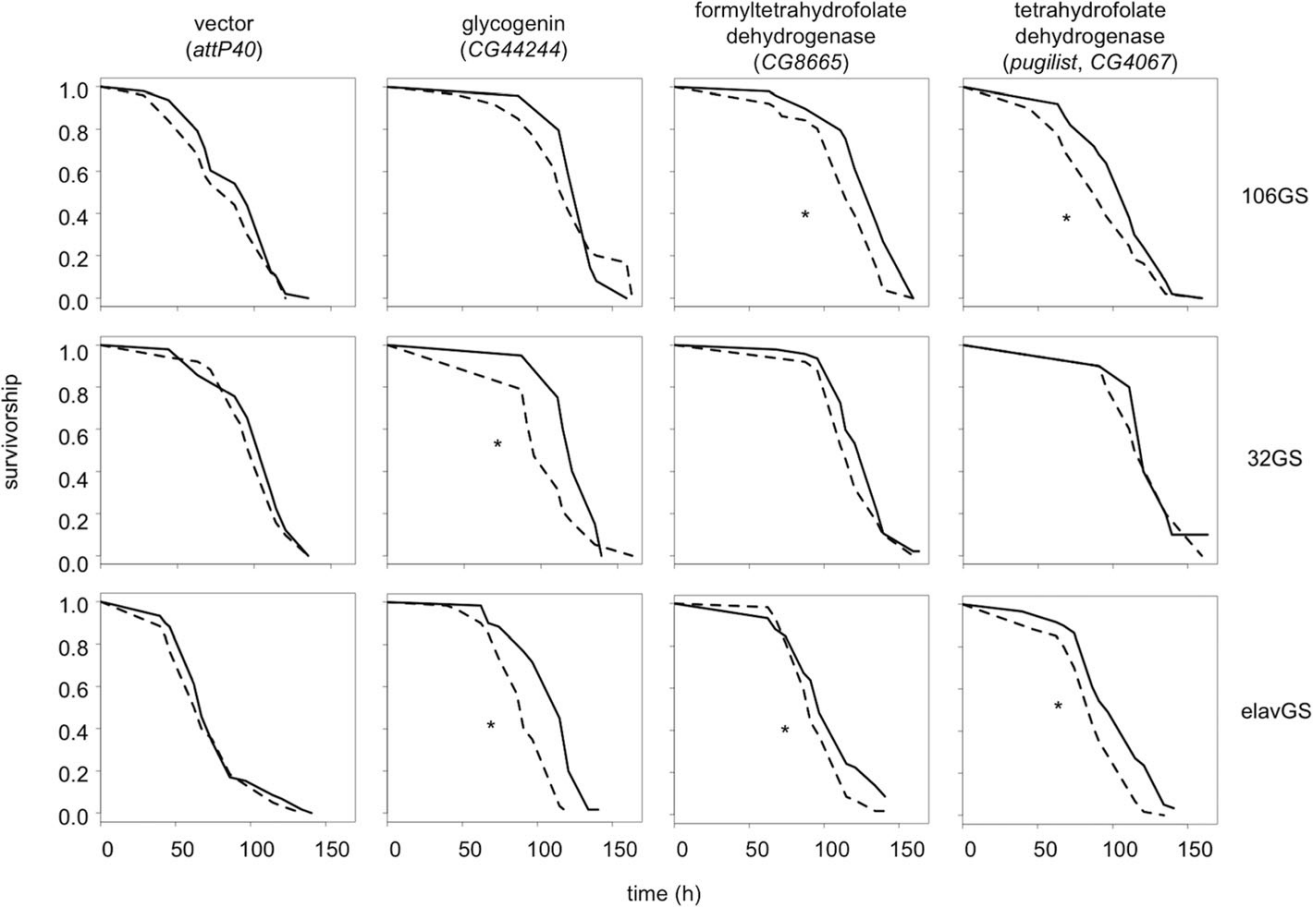
Knockdown of Genes in Glycogen or Folate Metabolism. F_1_ females from crosses of UAS-RNAi lines, targeting the indicated gene with either the GeneSwitch106 (106GS, top row), GeneSwitch32 (32GS, middle row), or elavGeneSwitch (elavGS, bottom row) drivers, were maintained on RU486 food (dashed line) or –RU control food (solid line) for 48 h prior to being transferred to H_2_O_2_ or control food. Survivorship was recorded for five replicate vials of ten flies per vial on H_2_O_2_ food and two to five replicates on control food (*n* = 20 or 5 flies). Asterisks indicate significant effect of RU486 (*P* < 0.05, log-rank test). Data are representative of one or two experiments, and the mortality of flies on control food (not shown) did not exceed 15% in any condition and did not depend on RU486 exposure

In addition to experiments with supplemental glycogen intermediates, we also used genetic manipulation to test for a role of glycogen metabolism in resistance to H_2_O_2_ food. The fat body is a site of glycogen storage in *Drosophila*, and RNAi targeting *glycogenin* (CG44244), the gene encoding the protein core of glycogen, in the fat body increased the sensitivity of flies to H_2_O_2_ food (Fig. 7). We found that knocking down *glycogenin* using the S32 fat-body driver reduced survivorship, while the S106 fat-body driver did not (Fig. 7). Glycogen is present in the *Drosophila* brain, and knocking down *glycogenin* with the neuron-specific elav GeneSwitch driver reduced survival on H_2_O_2_ substantially (Fig. 7) [47]. These data suggest that glycogen metabolism differentiates sensitive verses resistant flies.

### Folate metabolism

The folate pathway was also identified by *mummichog* among the features associated with H_2_O_2_ resistance and treatment (see Table S2, Additional File 2). The folate pathway is central to the synthesis of several amino acids, nucleotides, secondary metabolites and substrates for secondary modifications (e.g. methylation). *Mummichog* detected features that are consistent with metabolites both in the folate pathway as well as in peripheral pathways (e.g., *S*-adenosylmethionine), suggesting that the activity of the folate pathway differs between sensitive and resistant flies. To investigate the potential role of the folate pathway in H_2_O_2_ resistance, we tested the effect of metabolic gene knockdown on survival. Knocking down either CG8665, which encodes 10-formyltetrahydrofolate dehydrogenase (FDH), or *pugilist* (CG4067), which encodes tetrahydrofolate dehydrogenase (THFDH), in the fat body or in neurons reduced the survival of flies on H_2_O_2_ food (Fig. 7). In parallel experiments, RU486 pretreatment failed to affect survival in flies carrying the fat body S32 driver. These data suggest that folate metabolism in the abdominal fat body and neurons is important for survival on H_2_O_2_ food.

Supplemental folic acid was shown to reduce the levels of oxidative damage associated with knockdown of *parkin* in *Drosophila* neurons [48]. We also tested whether supplemental folic acid would affect survival on H_2_O_2_ food and failed to see a significant effect (data not shown).

## Discussion

### Dissecting variation for complex traits

Next-generation sequencing technology has provided geneticists with unprecedented power to identify single nucleotide variants associated with variation in complex traits in natural populations. But even with extremely large sample size (e.g., [20, 49, 50]), the percent of variance explained by SNPs in most studies remains small [51–53], and current models suggest that thousands of SNPs can contribute to any one trait [52, 54]. Our modestly powered GWAS failed to detect individual SNPs that explain a large amount of the variation in H_2_O_2_ resistance. However, our mixed model analysis estimates that approximately 34% of the variation in peroxide resistance could be explained by the additive effects of genetic variants within the DGRP. Similar to GWAS studies of other traits in this population, our results suggest that peroxide resistance is polygenic in the DGRP [28, 55, 56].

**Fig. 8:**
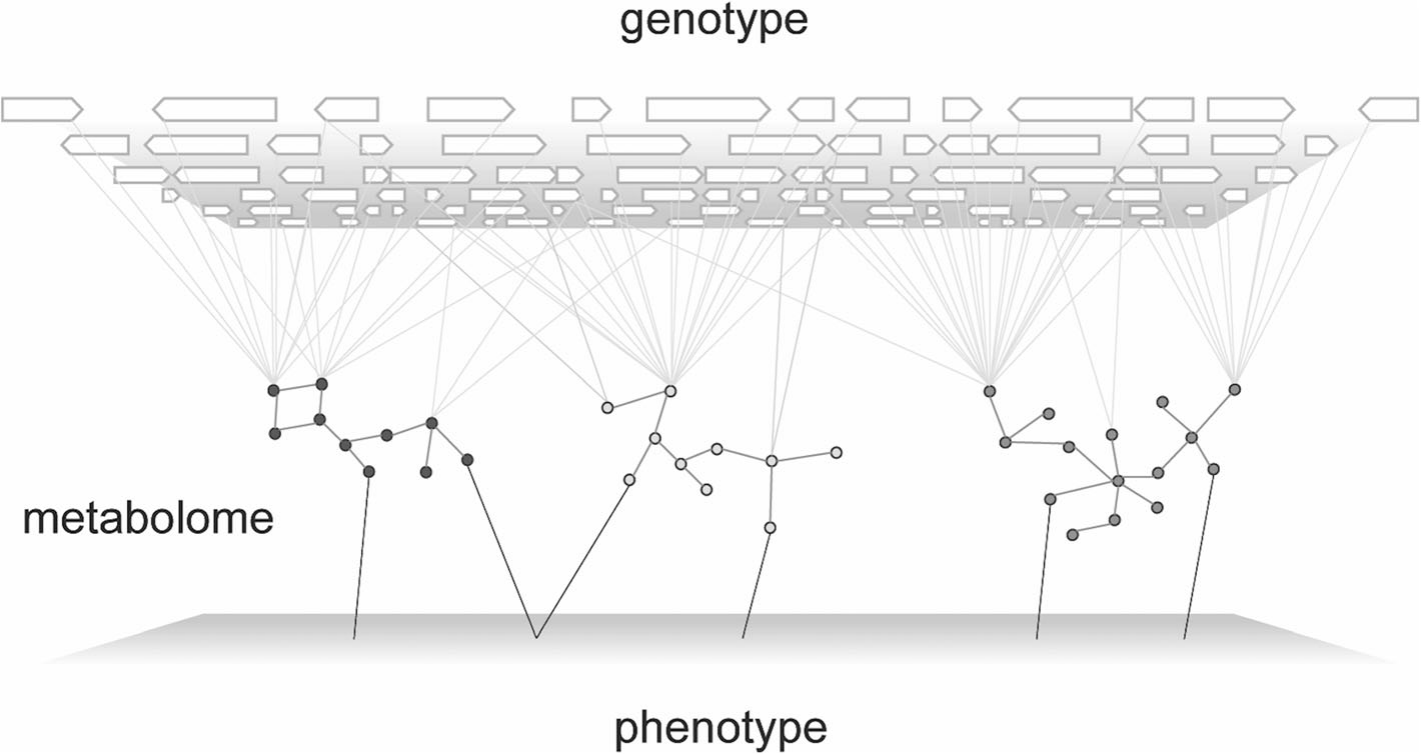
The Metabolome as a Bridge in the Genotype to Phenotype Map. Phenotypic variation, represented by the bottom plane, is influenced by the action of a large number of genes and genetic variants (upper plane) on a smaller number of metabolites or metabolic pathways. Direct influences on phenotype are shown as dark lines whereas indirect connections are shown with grey lines. Genetic variation can influence the levels of individual metabolites or the relationship between metabolites and, as a result, affect metabolic pathways that share direct connections to phenotype; or, genetic variation can affect phenotype more directly (not depicted). Two or more genes might act epistatically to influence the level of a single metabolite, and a single gene might act pleiotropically on multiple metabolites

The results presented here also point to the considerable potential of metabolic profiles to distinguish genetically determined phenotypic variation (Fig. 5), and moreover, to identify novel, causal molecular pathways associated with that variation. In light of our findings, we propose a model here whereby a large number of interacting genetic loci [57] converge through a more limited number of downstream metabolic pathways, which in turn make up the functional and structural building blocks of complex traits (Fig. 8).

### Metabolomic analysis

This study illustrates that untargeted metabolomic profiles, even those that include unknown chemical identities, give us tremendous power to 1) explain complex phenotypic variation; 2) identify novel pathways associated with this variation; and 3) in this particular study, suggest a novel hypothesis that resistance to stress might be caused by resistance of the metabolome to environmental perturbation.

#### The Metabolome as a biomarker

First, we find that the untargeted metabolome when compared to genotype associates more closely with phenotype. While a PCA of the metabolome clearly separates sensitive from resistant flies, a similar analysis of allelic variants among genotypes failed to clearly separate genotypes based on their survival on H_2_O_2_ food (Fig. 5). This contrast highlights the ‘proximity’ of the metabolome to trait on the genotype-phenotype map [4, 58].

Numerous other studies have shown metabolomic responses to diet, age, and temperature [32, 59–61], as well as body mass and body composition independent of diet [31], in diverse genotypes. However, these studies were not designed to test the relative association of genotype versus metabolome with phenotypic response. Moreover, studies that describe the effect of stress or environment on the metabolome often include only a single genotype and this may fail to resolve the systems-level association between genotype, environment, metabolome and phenotype [62–65]. In light of our results, future studies would benefit from a clearer characterization of the power of the metabolome relative to the genome to distinguish biologically relevant phenotypic variation.

#### Stress resistance pathways identified by metabolomics

Second, our metabolomic profiling suggests several possible mechanisms that underlie the phenotypic variation observed here. As we emphasize above in presenting the results, and discuss further here, in *Drosophila* studies, variation in the ability to survive oxidative stress could be confounded with starvation resistance. This is important to keep in mind as we discuss possible mechanisms that underlie the phenotypic variation seen here. Nonetheless, the ability of the metabolome to distinguish resistant and sensitive phenotypes is notable.

Several studies have used metabolomics to study the effects of oxidative stressors in *Drosophila* in a single genetic background. For example, paraquat treatment alters branched-chain amino acid, starch/sucrose, and fatty acid metabolism in the *Drosophila* brain [65]. We also detect significant effects of H_2_O_2_ food on the first two of these three pathways (see Table S2, Additional File 2). The lack of evidence for an effect on the third pathway, fatty acid metabolism, is perhaps not surprising, given that our analysis was limited to aqueous metabolites. There are two important caveats to this earlier work as well as to the present study. First is the ambiguous nature of the untargeted metabolite data. For many features in the global metabolome profiles, we know mass/charge ratios but not chemical structure. Second, given the relationship between resistance to H_2_O_2_ and starvation (Fig. 1), we expect that the metabolites associated with exposure to H_2_O_2_ or paraquat might relate to nutrient intake and/or storage, rather than oxidative stress alone. This confounding influence of H_2_O_2_ on feeding could explain the results of a variety of studies that use survival on food supplemented with H_2_O_2_ as a measure of oxidative stress resistance.

One way to disentangle the effects of oxidative stress from a secondary effect of a stressor administered in the diet on nutrient update is to genetically manipulate endogenous levels of reactive oxygen species. Two recent studies compared the metabolome of flies with null mutations in superoxide dismutase (*sod*^*−*^) to that of flies either chronically or acutely exposed to paraquat-supplemented diet [62, 63]. Each of these conditions had a distinct effect on the metabolome, with the *sod*^***−***^ mutant metabolic profile clearly separating from the other conditions in unsupervised clustering [63]. Our analysis suggests that at least some of the differences between the *sod*^*−*^ and paraquat-treated metabolome could be due to altered feeding or starvation in paraquat-treated flies.

Keeping these caveats in mind, we find strong evidence for two pathways associated with H_2_O_2_ resistance- glycogen metabolism and folate metabolism. Indeed, the apparent decrease in glycogen content in flies sensitive to H_2_O_2_ compared to control food suggests that sensitive flies are exhausting their glycogen pool in response to stress, which is consistent with previous studies of flies on food with paraquat or under starvation [36, 66, 67]. Maltodextrins are intermediates in the glycogen pathway, which is used to store and retrieve glucose for the glycolysis and pentose phosphate pathways (Fig. S5). We found that supplemental maltose or glucose enhances survival on H_2_O_2_ food for all genotypes tested in our study and that RNAi targeting *glycogenin* either in the fat body or in neurons reduces survival on H_2_O_2_ food (Figs. 6 and 7).

Interestingly, knockdown of *glycogenin* affected survival, but only with the S32 driver, which is expressed primarily in the head fat body, and not the S106 driver, which is expressed in the abdominal fat body. This suggests that *glycogenin* functions in the head fat body to mediate resistance to H_2_O_2_ food [68, 69]. However, we cannot rule out the effect of other differences between the knockdown of *glycogenin* by S32 compared to the S106 driver.

*Mummichog* also detected enrichment of the folate pathway (see Table S2, Additional File 2). Several lines of evidence from this work and previous studies indicate a role for the folate pathway in stress resistance in *Drosophila* [25, 48, 70–75]. We show that knocking down either the folate pathway genes *pugilist* or CG8665 in either the fat body or neurons reduces survival on H_2_O_2_ food while not affecting survival on food without H_2_O_2_ (Fig. 7 and data not shown). Several studies find that transcripts encoding enzymes of the folate pathway are up-regulated following either administration of paraquat or H_2_O_2_ in food, over-expression of manganese superoxide dismutase, or in mutants with mitochondrial dysfunction [72–77]. Additionally, two missense variants in *pugilist* associate with paraquat resistance in the DGRP [25]. Functional studies show that overexpression of *Drosophila nmdmc*, which encodes methylenetetrahydrofolate dehydrogenase, enhances resistance to paraquat [71], and that folic acid supplementation reduces oxidative damage to lipids and endogenous H_2_O_2_ levels associated with knockdown of *parkin* in *Drosophila* neurons [48]. While our metabolomic data and previous genetic studies [70] point to a role for folate metabolism, in our own GWAS analysis, neither *pugilist* nor CG8665 were clearly associated with survival on H_2_O_2_ (*P*_*min*_ > 0.002 in both cases).

#### Adaptive shifts versus robustness of the metabolome under stress

Third, it appears that the metabolome responds to the presence of H_2_O_2_ more strongly in sensitive than in resistant lines (Figs. 4 and S4). One might have expected the opposite pattern, whereby greater resistance is associated with an *adaptive* response of the metabolome to an environmental stressor (e.g., [78–80]). Instead, it appears that resistance in this study is associated with the ability to maintain the metabolome in its current state. This observation is made possible by having multiple sets of stress-resistant and stress-sensitive genotypes. We interpret these results as suggesting not only that resistance to H_2_O_2_ food is explained by the metabolome, but also that the metabolome is more *robust* in resistant genotypes, being less likely to change when faced with a stressor. We do not propose that metabolic robustness is a universal or causal feature of stress resistance, though this is an interesting hypothesis to test, but our data suggest that metabolic robustness and/or resilience might contribute to phenotypic variation in nature.

This observation, combined with the fact that the metabolome associates with phenotype while the genotype does not, suggests the hypothesis that there are genetically diverse ways to achieve resistance to H_2_O_2_, but that these diverse genetic paths converge at a common metabolome associated with resistance. In future studies it would be worth asking if, across a broad range of phenotypes, there is a correlation between stress resistance and the ‘resistance’ of the metabolome to stress-induced alteration, or vice versa for traits that require metabolic adaptation.

### Genomic analysis

We studied survival on H_2_O_2_ food in the DGRP, a population representing a sample of natural genetic variation. We estimate broad-sense heritability for mean lifespan under H_2_O_2_ at *H*^2^ = 44.8% based on the within-genotype variance in phenotype compared to total phenotypic variation. There are significant caveats to estimating heritability in the DGRP, including the relatively small number of lines and the potential to overestimate the degree of heritability due to the low among-line environmental variance associated with repeated measures of biological replicates within inbred genotypes. With these caveats in mind, we estimate ${{\hat{h}}^2}_{SNP}$ of 34.2%. This estimate is slightly higher than the narrow sense heritability *h*^*2*^ estimated for starvation resistance in other studies in the DGRP using similar methods, although estimates of *h*^*2*^ in the DGRP vary widely [56, 81]. Given that the DGRP is highly inbred, we have not empirically evaluated the extent of additive gene action in our study. However, our results indicate that genetic variation contributes significantly to H_2_O_2_ resistance in the DGRP.

Mapping variants associated with mean survival revealed 14 SNPs that were significant and together these variants define 7 loci (Fig. 2). We limited our analysis to variants passing MAF and missing genotype filters among 179 DGRP lines. While the DGRP incorporates substantial allelic diversity from the wild, it is a population of inbred lines with very low levels of heterozygosity compared to its parent wild population and that has also been purged of deleterious alleles of strong effect [55]. Along with the missing genotypes there are also uncharacterized structural variants within this population [22]. For these reasons, we do not expect this study to identify all variants in the DGRP associated with survival under H_2_O_2_ stress, nor their mode of gene action. Similar to other studies with the DGRP, we failed to find common alleles with large and highly significant effects, suggesting that variation in survival on H_2_O_2_ food is influenced by many loci of small effect [55, 56]. Importantly, in the relatively small population used for metabolomics, neither clustering nor multivariate analysis of genotypes associated with the discrete resistance trait whereas similar analyses of metabolome data did distinguish resistant and sensitive genotypes (Fig. 5).

Although we hoped to pinpoint specific genes that influence H_2_O_2_ resistance, we face the statistical challenge that among the DGRP lines we studied, the number of variants per gene ranged from 1 to 4490 with a mean of 237. To overcome the increased risk of false positives in genes that contain a large number of polymorphisms, we used a permutation approach to measure association between genes and phenotype. Our approach, like other methods, comes with caveats, one being the imperfect annotations of variants and genes. We have limited this analysis to only those variants associated with the FlyBase gene annotation, including 1 kb upstream and 1 kb downstream of the primary transcript [22]. Intergenic variants might affect the expression of trait-associated genes. However, we did not attempt to account for those effects in this study. Additionally, variants that are associated with candidate genes can instead exert their effect on phenotype by modifying the expression of other local genes, rather than the candidate gene, and this would result in misattributing the significance to the gene containing the variant rather than the real trait-associated gene [82].

#### Stress resistance pathways identified by genetic association

Our genomic analysis led to several interesting and potentially related genes associated with H_2_O_2_ resistance, including *ush, NPF* and the pickpocket paralogs *ppk7* and *ppk14*. Each of these genes is implicated in several processes and though we do not rule out causal associations between these processes and resistance to H_2_O_2_ food, based on published studies, we argue that genetic variation in these genes influences feeding and/or metabolism to explain their effect on H_2_O_2_ resistance (Fig. S7).

As we show, flies on H_2_O_2_ food substantially reduce food intake, and their lifespans appear to be a function of starvation (Fig. 1). Interestingly, feeding behavior is controlled by neuronal signaling involving several of these candidate genes. Larvae require *NPF* to signal the intake of noxious food under starvation conditions [83], and NPF-expressing neurons in adults couple hunger to memory performance [84]. Interestingly, IlS in neurons expressing the NPF receptor represses larval feeding, suggesting that NPF controls feeding through neuronal IlS [83]. We postulate that the candidate *ush* influences survival on H_2_O_2_ food as a peripheral regulator of IlS [43]. This appears to be a function directly attributed to the USH protein, as its human homolog FOG has been shown to directly bind and inhibit the PI3K complex [43]. Inactivation of PI3K leads to depletion of nutrient stores in the fat body and its constitutive activation reduces both nutrient stores and survival under starvation [85]. PI3K is also a negative regulator of FOXO in the IlS pathway, which has well characterized roles in the response to starvation and to oxidative stressors in food [68, 86–89]. Interestingly, ablation of insulin-like peptide-producing cells in *Drosophila* increases survival both on food containing paraquat and under starvation, alters whole body levels of glycogen, and leads to misregulation of metabolic genes in the glycogen pathway, which could link the effect of NPF and *ush* polymorphism to the variation we see in glycogen metabolism [90, 91].

The effects of two other candidates, *ppk7* and *ppk14*, might also be linked to feeding and metabolism through their potential role in nutrient signaling. *ppk7* and *ppk14* are members of the pickpocket gene family that are expressed in neurons and signal taste cues, modulate feeding, and may influence energy metabolism [92–95]. Interestingly, *Drosophila ppk28* was recently shown to interact with glucagon-like hormone (AKH) signaling, a pathway involved in regulating glycogen metabolism in flies [91, 96]. Together, the genetic analysis of H_2_O_2_ resistance has revealed pathways whose role in survival could be explored in future studies.

### Starvation as a confounding factor in stress assays

Our study used H_2_O_2_-supplemented food as the stressor. Oxidative stressors such as H_2_O_2_, paraquat, or menadione are often administered to *Drosophila* by supplementing the diet, and each of these treatments dramatically reduces survival in a dose-dependent manner [25, 87]. After screening the DGRP for survival on H_2_O_2_ food, we noticed that these survival times correlated closely with survival times under both paraquat and menadione exposure, and even more so with survival times measured under starvation [26].

Feeding is essential to the survival of adult *Drosophila*, and feeding behavior is influenced by a variety of cues, including food acidity or the presence of bitter compounds, hypoxia, and the nutrient content of food [97–100]. While some stressors affect the preference for food of particular composition [98, 101], others may alter feeding behavior by affecting satiety [97]. We show that flies consume very little food containing 2% H_2_O_2_. This effect is not limited to H_2_O_2_, as many supplements have been found to reduce feeding in flies [97], and this may extend to other oxidative stressors including paraquat and menadione. While several studies have detected reduced feeding in response to paraquat-containing food [102, 103], others detected no difference [67]. Contrary to the latter study, our data suggest that feeding is significantly reduced in the three genotypes tested in assays that measured feeding over either 2 h or 24 h (Fig. 1). Several differences in the experimental setting may explain the discrepancy between this study and Riahi et al. (2019) [67].

It is possible that the effect we see relates to oxidative stress and not an aversion to food supplementation, as deviation from normoxia alters feeding behavior of *Drosophila* larvae in a manner that appears to rely on H_2_O_2_-sensitive neurons [104]. Also, the UV light-avoidance of egg-laying females appears to signal through H_2_O_2_-sensitive taste receptors in *Drosophila* [105], and H_2_O_2_ also inhibits feeding in *Caenorhabditis elegans* through taste receptors [106]. Additionally, two recent studies found contradictory roles for the histone methyltransferase G9a in survival in response to oxidative stressors and starvation, indicating that environment and genetic background may affect stress-response in *Drosophila,* but also suggesting that starvation and oxidative stress resistance may share underlying biological pathways [36, 67]. These studies suggest that susceptibility to oxidative stress and starvation are partially separable. However, they do not rule out a main effect of H_2_O_2_ on survival due to starvation. The relationship between lifespan under starvation and survival on H_2_O_2_ food has implications for studies that draw conclusions about stress resistance in response to agents administered in food. The effects of altered feeding patterns or nutrient deprivation should be accounted for when analyzing the effects of stressors or drugs administered in the diet.

## Conclusions

### Genetic variation in a complex trait converges on the metabolome

The sample of genotypes in this study show a consistent metabolic signature associated with their phenotype. Thus, a potentially wide degree of genotype space may converge on a smaller number of metabolic pathways to shape phenotype.

### Metabolome robustness associates with stress resistance

Contrary to the metabolic change that might be expected in animals resisting stress, we find that the metabolome of resistant animals appears robust to stress treatment. This suggests that maintaining metabolism in the presence of certain stressors is a means of survival.

### Glycogen and folate metabolism and several genes involved in nutrient signaling mediate resistance to peroxide food

Genetic and metabolomic analysis of peroxide resistance revealed roles for glycogen and folate metabolism and genes with known roles in nutrient signaling. Future studies to understand this network may reveal novel mechanisms of stress resistance.

### Starvation explains the lifespan response to peroxide food

*Drosophila* are sensitive to diet, including additives and food-borne treatments. We show that the response to H_2_O_2_ in food can be explained by starvation. These effects may dramatically confound assays that examine responses to treatments delivered by supplementing the *Drosophila* diet.

## Methods

### Genetic stocks

*Drosophila* Genome Reference Panel (DGRP) lines were obtained from the Bloomington *Drosophila* Stock Center (BDSC). Genes encoding enzymes involved in the glycogen or folate pathways were identified using the Kyoto Encyclopedia of Genes and Genomes database (http://www.genome.jp/kegg/pathway.html). The expression of GWAS candidates or genes encoding enzymes in the glycogen or folate pathways were manipulated using the GAL4 GeneSwitch/UAS system [44]. The drivers S106 (BDSC #8151), S32 (BDSC #8527) or elav GeneSwitch (BDSC #43642) were crossed to flies carrying UAS-RNAi transgenes targeting candidate genes: *ush* (CG2762, BDSC #32950 (*ush_44*) and BDSC #44014 (*ush_57*)), *Glycogenin* (CG44244, BDSC #42565), CG8665 (BDSC #62266), and *pugilist* (CG4067, BDSC #42950), or the attP2 background control (BDSC #8622). The *NPF*^*SK1*^ allele is a 179 bp deletion within the coding region created by CRISPR [107]. *NPF*^*SK1*^ was backcrossed at least six times into the Canton-S background prior to testing. Stocks were maintained on standard cornmeal-sugar-yeast food at 25 °C on a 12/12 h light/dark cycle at 50–70% RH.

### Media

*Standard food* was made by cooking 12 g Drosophila agar (type II, Genesee Scientific, El Cajon, CA), 25 g brewers yeast (MP Biomedicals, Solon, OH), 55 g glucose monohydrate (MP Biomedicals), 30 g sucrose, 60 g corn meal, 3 g methylparaben (Genesee Scientific). 12 g 100% ethanol (Decon Labs, King of Prussia, PA), and 3 g propionic acid (Fisher Scientific, Pittsburg, PA) per liter of water. A small amount of dry active yeast was sprinkled onto standard food prior to use.

*Peroxide food* was made in one of two ways, for 2% food, agar was melted into 2% glucose monohydrate and 0.3% propionic acid and, after the food had cooled to less than 60 °C, 30% H_2_O_2_ (Fisher Scientific) was added to reach 2%, or the same volume of water was added for the control food. For the 3% H_2_O_2_ food, the recipe was the same with the exception that 30% H_2_O_2_ was added to reach 3% H_2_O_2_. Approximately 5 mL of food was dispensed into 25 mm wide × 95 mm tall polystyrene vials.

*Starvation food* was made by melting 2% agar into 0.3% propionic acid and dispensing into vials.

*Food supplemented with carbohydrates* was made by adding 2% of either D-(+)-maltose, β-lactose (both from Sigma, St. Louis, MO), or additional glucose to the 2% glucose control food.

*RU486 food* to induce the GAL4 GeneSwitch system was made by overlaying ~ 5 mL standard food with either 50uL of 25 mg mL^− 1^ RU486 (mifepristone, Cayman Chemical Company, Ann Arbor, MI) dissolved in 100% ethanol or the same volume of 100% ethanol alone for the -RU control food. Ethanol was allowed to evaporate overnight at 22 to 24 °C prior to using the food.

### Genetic manipulation

F_1_ GAL4/UAS flies were collected over four days (day 0 to 3). These flies were allowed to mate for 24 h, at which time they were anesthetized and sexed, and females (ten per vial) were then allowed to feed for two days on RU486 or -RU food. After 48 h on RU486 or -RU food, flies were transferred without anesthesia to H_2_O_2_ or control food to measure survival. Negative genetic controls included F_1_ GAL4/attP flies which were crosses of the GAL4 driver to either the attP2 or attP40 lines from the Transgenic RNAi Project collection, where attP is the empty P-element docking site for the UAS transgenes (http://fgr.hms.harvard.edu/fly-in-vivo-rnai). Negative genetic controls were raised, induced and assayed in parallel with experimental flies.

### Stress survival assays

To measure the variation in resistance to oxidative stress across a lab population, we measured the survival of mated females from 179 DGRP lines in a multi-block design on H_2_O_2_ food [26]. For each block, flies were raised under low-density conditions by allowing ~ 50 flies to lay eggs for one day on standard food in bottles. Flies for the assay were then collected over two or three days and then allowed to mate for 24 h on standard food. Four vials of flies on H_2_O_2_ food and one or two vials of flies on control food without H_2_O_2_ were included for each genotype in each trial. Each vial contained 10 mated females. Control vials without H_2_O_2_ were included to confirm that mortality was due to H_2_O_2_. In knockdown experiments, we included 5 to 8 H_2_O_2_ vials and 5 to 8 control vials to ensure that any effect of gene knockdown on survival in the absence of H_2_O_2_ could be measured. To measure line weights, 24 h after beginning a lifespan assay, flies from an extra control food vial were frozen at − 80 °C and were later collectively weighed on a microbalance (XS105, Mettler Toledo, Columbus, OH). In all blocks, dead flies in each vial were recorded two to four times per day using D-life software until all H_2_O_2_-treated flies had died [23]. For assays involving the Drosophila Activity Monitor System (DAMS, TriKinetics Inc., Waltham, MA), the activity of 38 to 48 individual flies per genotype was recorded simultaneously every minute over the experiment.

All calculations were performed in R [108]. Mean lifespan was estimated from H_2_O_2_ assays using the restricted mean (default settings) in the Kaplan-Meier model with the survival package [109]. We ran 17 blocks with a mean of 14.5 lines (range = 4 to 35 lines) per block. We used 2% H_2_O_2_ food for the first 10 blocks and 3% H_2_O_2_ was used for the last seven blocks. The switch between 2 and 3% H_2_O_2_ was made accidentally and was realized after the conclusion of the study. We used the following mixed model in the R package lme4 to test for an effect of these two food treatments on the log of mean lifespan:

$$ \kern0ex lo{g}_e lifespan= food+ weight+\left(1| block\right)+\left(1| line\right)+\varepsilon $$

where food (2% or 3%) and weight were fixed effects, and block and genotype were both random effects along with the error term ϵ. The significance of random effects was assessed by the likelihood ratio test, and of fixed effects by ANOVA. We found no difference between 2 and 3% H_2_O_2_ doses on lifespan (*β* = − 9.1 × 10^− 4^, *P* = 0.988).

To compare lifespans on H_2_O_2_ food to lifespans during starvation, 2 to 5 replicates of twenty 3-to-5 day-old mated females were assayed using D-life on agar food either with or without 2% glucose (see Media). The lifespan of each line on H_2_O_2_ food was measured twice in separate trials for this comparison, while the lifespan under starvation was measured once. To measure the effect of supplemental carbohydrates on lifespan ten replicates of ten 1-to-3 day-old mated females per genotype were allowed to feed on supplemented food for four days and then transferred without anesthesia to 2% H_2_O_2_ or control food to assay survival.

### Feeding assays

To measure feeding rate, we used both dye incorporation and CAFE assays. For both assays, flies were allowed to mate for 24 h and then separated sexes over light CO_2_ anesthesia and transferred to agar-only food for 24 h of starvation. For dye incorporation, after starvation, flies were immediately transferred without anesthesia into vials that contained either H_2_O_2_ or control food with 2.5% FD&C Blue Dye #1 (Spectrum Chemicals, Gardena, CA). After 2 h on dye-containing food, flies were flash frozen in liquid N_2_, homogenized in water, centrifuged at 16,000rcf for 1 min, and the absorbance of the supernatant was measured at 630 nm. The absorbance of each sample was normalized by dividing by the number of flies in the sample (*n* = 7 to 11 flies per sample).

For the CAFE assay, ten replicates of 10 mated females were starved for 24 h and transferred without anesthesia to assay chambers. Assay chambers were 15 mL conical bottom polystyrene tubes (Corning Inc., Corning, NY) containing water under a foam partition to maintain humidity but not allow flies to drink, and fitted with a 0.75 mm ID glass capillary (World Precision Instruments, Sarasota, FL) which had been filled with 2% glucose, 0.3% propionic acid supplemented with either 2% H_2_O_2_ or water. Flies were housed in the assay chambers at 25 °C on a 12/12 h D/L cycle in an incubator at 60–70% RH for 24 h before the volume of food consumed was assayed by measuring the difference in height of the top of the liquid food in the capillary and multiplying by π·0.375mm^2^.

### Genetic analysis

We estimated broad-sense heritability of fly lifespan within each of the seven blocks in which at least 13 randomly chosen DGRP lines were included, treating each block as an independent measure of heritability. For each block, heritability was estimated by: *σ*_*L*_^2^ / (*σ*_*L*_^2^ + *σ*_*E*_^2^) in an ANOVA, where *σ*_*L*_^2^ is the among-line variance in the weight-residuals of lifespan and *σ*_*E*_^2^ is the average within-line variance [110]. Mean heritability across blocks and its standard error were calculated from these seven estimates. We estimated SNP heritability (${\hat{h}}_{SNP}^2$) using genetic variance ( *σ*_*g*_^2^) and the residual variance ( *σ*_*e*_^2^) estimated by restricted maximum likelihood in the NAM package [54, 111] using the model

$$ {\log}_e lifespan={X}_b+K+\varepsilon $$

where the lifespans were from the 9988 individuals across the study, *X*_*b*_ is the design matrix of fixed effects, which include block, mean fly weight, *Wolbachia* status and the genotype at the four segregating inversions whose MAF was at least 3%: *In(2 L)t, In(2R)NS, In(3R)P* and *In(3R)Mo*. *K* is a genetic relationship matrix made with 712,878 LD-pruned variants (*r*^*2*^ < 0.5), MAF ≥5%, genotype call rate ≥70%, in PLINK according to [112] and is modeled as a random effect, and *ε* is the error term.

For GWAS, we used residuals from linear regression of lifespan versus weight as our measure of H_2_O_2_ resistance. To correct for block effects, weight residuals of lifespan were centered (mean = 0) and scaled to unit variance (SD = 1) by block. For lines measured in more than one block, we calculated the average block-centered lifespans across blocks. We used stepwise regression in the MASS package to identify significant covariates among the chromosomal inversions and *Wolbachia* status [22]. *In(2 L) t* was the only significant covariate (*P* = 0.0277, ANOVA) and residuals from the linear regression of H_2_O_2_ resistance on *In(2 L) t* genotype were used in a linear model of SNP-phenotype associations in PLINK [40]. Approximately 1.93 million SNPs with a MAF ≥0.05 and <30% missing genotypes were tested for association with H_2_O_2_ resistance from 179 DGRP lines. To account for population structure, we used the Tracy-Windom test in the AssocTests package to evaluate eigenvalues from 20 PCs of genotype, and retained the first four PCs as covariates in the model (α = 0.05, [113]). Genome-wide significance was determined by controlling for FDR at 0.2 using the *q* value method [41].

Gene-level associations with H_2_O_2_ resistance were derived using two rounds of permutation to reduce computational burden and to estimate more precise empirical *P* values. The first round tested 4810 genes that had at least one variant associated with phenotype with *P* ≤ 0.01, thus avoiding computation of gene-level statistics for genes with a very low chance of being significant. We refer to SNPs within 1 kb upstream and 1 kb downstream of the gene model in FB release 5.49 as associated with that gene [22, 114]. In the initial analysis, 10,000 permutations of phenotype were performed, and the association between phenotype and each SNP in a gene was tested using the same linear model employed for SNP-phenotype associations described above. An initial empirical one-tailed P value for each gene (*P*_*gene*_) was calculated by comparing the maximum test statistic (T_max_) among the SNPs in each gene from the real GWAS to the T_max_ for each of 10,000 permutations. A second round of selection was then run, this time choosing only genes with *P*_*gene*_ ≤ 0.01 from the first round. The 192 genes with initial *P*_*gene*_ ≤ 0.01 were then subjected to 1 million more permutations and the resulting *P*_*gene*_ values were used as our measure of gene-trait association. We use *q* values to estimate the FDR for each *P*_*gene*_. Over-representation by biological process and pathways was tested using Fisher’s exact test in PANTHER (version 13.1) and the GO-slim subset of biological processes and PANTHER pathways among the 13,767 gene models in *Drosophila melanogaster* [115].

### Metabolomic analysis

Eight of the resistant and eight of the sensitive DGRP lines were selected based on their lifespans and line weights to reduce the effect of fly size on resistance. Lifespan for these lines on H_2_O_2_ food was again measured in a single block, and 24 h after exposure to the H_2_O_2_ or control food, 3 replicates of 5 flies each were collected, flash frozen in liquid nitrogen, and then stored at − 80 °C. Each *Drosophila* sample was weighed and then homogenized in 200 μL water with PBS in a microfuge tube immersed in an ice bath. Methanol (800 μL) was then added, followed by vortexing for 2 min and incubation at − 20 °C for 30 min to precipitate proteins. Samples were sonicated in an ice bath for 10 min and then centrifuged at 17,000 rcf for 5 min at 4 °C. From each tube, 900 μL supernatant was transferred to a new microfuge tube for drying under vacuum at 30 °C (~ 3 h). The completely dried samples were reconstituted in 100 μL 40% water/60% HPLC-grade acetonitrile (ACN, Fisher Scientific) for liquid chromatography-mass spectroscopy (LC-MS) analysis. A pooled quality control (QC) sample was made by combining ~ 5 μL aliquots from each reconstituted sample. The QC was analyzed once for every ten study samples to serve as a technical replicate throughout the data set to assess process reproducibility and allow for data normalization to account for any instrument drift.

LC-MS analysis was performed using an LC-QTOF-MS system (Agilent Technologies, Santa Clara, CA) consisting of an Agilent 1200 SL liquid chromatography system coupled online with an Agilent 6520 time-of-flight mass spectrometer. A 5 μL aliquot of reconstituted sample was injected onto a 2.1 × 150 mm Waters BEH-Amide 2.5 μm particle column at 35 °C. The metabolites were gradient-eluted at 0.3 mL/min using mobile phase A, 5 mM ammonium formate (Sigma) and 0.0125% formic acid (Sigma) in 97% water/3% ACN, and mobile phase B, 5 mM ammonium formate and 0.0125% formic acid in 3% water/97% ACN (98% B for 1 min, 98 to 77% B in 6.5 min, 77 to 39% B in 4.5 min and 39% B for 7 min). The MS interface capillary was maintained at 325 °C with a nebulizing gas pressure of 45psig, and a drying gas flow of 9 L/min. The capillary voltage for positive ion injection was 3.5 kV.

Data from 60 to 1000 m/Z was acquired using Agilent MassHunter Workstation data acquisition software B.02.01 (B2116.30) in centroid mode with a threshold of 200 or 0.01%. LC-MS data was processed using XCMS online (version 2.2.5) and a list of ion intensities for each detected peak was generated using a retention time index and m/z data as the identifiers for each ion (e.g. M62T11, for median m/z 62 and median retention time rounded to 11 min). Data are reported at Level 4 of the Metabolomic Standards Initiative, with annotation of these features as unknown compounds conservatively limited to exact mass and retention time [116].

Global LC-MS provided measures of 3028 features with 2.4% missing data from positive mode, and 2921 features with 2.2% missing data from negative mode in a total of 93 samples. All the features with ≥5% missingness and with ≥30% coefficient of variation (CV) in QC samples were excluded, leaving 2722 and 2691 features in positive and negative modes, respectively. We imputed the remaining missing values using the K-nearest neighbors imputation method implemented in the R impute package [117]. The log_2_-transformed feature abundance was median normalized prior to imputation. For the unsupervised learning, the batch effect and sample weight were removed using the limma removeBatchEffect function prior to the PCA and clustering analysis [118].

To examine the effect of trait and treatment interactions on metabolite features we fit a linear model to the imputed data to detect the group differences (e.g. resistant H_2_O_2_ vs. resistant Control, sensitive H_2_O_2_ vs. sensitive Control, resistant H_2_O_2_ vs. sensitive H_2_O_2_, resistant Control vs. sensitive Control, and trait and treatment interaction) using the Bioconductor limma package, while adjusting for the batch effect (e.g. block) and sample weight as covariates in our model. We used the Benjamini-Hochberg multiple testing method to control the FDR and selected metabolites at an FDR of 10% [119].

To identify metabolic pathways whose activity could explain the distribution of m/z and retention times among the LC-MS features associated with trait differences within each treatment, or treatment differences within each trait, or the interaction between the treatment and trait, we used *mummichog* version 1.0.10 [46]. *Mummichog* searches known metabolic pathways for potential enrichment among m/z and retention time data. In this case, *mummichog* was provided sets of features which showed significant effect from the linear model described above. The BioCyc *D. melanogaster* (version 16.5) metabolic model was used as the source of pathway data. One hundred permutations of the data were performed by *mummichog* to estimate the null distribution.

## Supplementary information

**Additional file 1: Table S1.** Variants Associated with Peroxide Resistance all 14 SNPs associated with peroxide resistance below FDR = 0.2. Annotations are based on gene models from FlyBase version 5.48 and SNPs more than 1 kb from an annotated gene are assigned NA. Asterisks indicate SNPS associated with more than one gene.

**Additional file 2: Table S2.** Summary of Mummichog Analysis. Description: Metabolic pathways identified by mummichog from the features detected in either positive or negative LC-MS (mode) that were significant from group comparisons (e.g., resistant H_2_O_2_ vs. resistant Control, sensitive H_2_O_2_ vs. sensitive Control, resistant H_2_O_2_ vs. sensitive H_2_O_2_, resistant Control vs. sensitive Control, and interaction) in the linear model. BioCyc pathways (pathway) were identified, and the significance of enrichment was assessed, in part by the number of LC-MS features in a given pathway (overlap_size) and the size of the pathway (pathway_size), by permutation (*n* = 100) to give a *P-*value.

**Additional file 3: Table S3.** Summary of XCMS metabolite feature data. Description: Untargeted LC-MS features analyzed in this study. Features were given names (e.g. M438T2) based on their median mass-charge ratio (mzmed) and median retention time, in minutes (rtmed). The minimum (mzmin) and maximum (mzmax) mass-charge ratios for each feature are given, along with their minimum and maximum retention time (rtmin, rtmax, respectively). When a tentative identification was made by XCMS, the matching compound (id) is given along with the matching ion (match_form) and the difference in mass (mz_difference) between the LC-MS feature and the matching ion. These data are from positive and negative modes (mode), each data mode was analyzed separately and combined on this table.

**Additional file 4: Figure S1.** Trait Correlation Analysis within the DGRP. The correlations of peroxide lifespan (scaled) with several other traits are shown. The traits include lifespan during exposure to starvation [26, 37], or to the log-transformed lifespans during exposure to oxidative stressors paraquat or menadione bisulfite (menadione) [25]. Least-squares regressions are shown in red. Above the diagonal are Pearson’s correlation coefficients for each pair of traits. There is significant correlation for peroxide survival with each of the traits shown (*P* < 0.05, corrected for multiple comparisons).

**Additional file 5: Figure S2.** Permutation Approach Corrects for Bias in Gene-Level Associations (a) *–log*_*10*_ P_min_ for 4810 gene models plotted over the number of variants per gene. (b) *–log*_*10*_ P_gene_ for 533 gene models that were permuted 1 million times plotted over the number of variants per gene. The red lines in (a) and (b) are cubic splines using the default parameters in the R function smoothspline.

**Additional file 6: Figure S3.** Lifespan and Fly Mass and the Selection of Lines for Metabolomics Mean lifespan (*n* = 17 to 80 flies) for each DGRP line over the average fly mass (*n* = 5 to 25 flies). (a) Linear modeling found a significant interaction between mean lifespan from all blocks and fly mass (*P* = 1.6 × 10^− 5^). Mass remained significant across the study after correcting for block effects (*P* = 7.5 × 10^− 6^). (b) The lifespans of lines chosen for metabolomics minimized the effect of mass on lifespan (*P* = 0.171).

**Additional file 7: Figure S4.** Projections of Metabolomic Principal Components The first and second principal components of the positive mode data labeled to indicate resistant and sensitive genotypes, as well as the effect of treatment (control vs. H_2_O_2_). Trait and treatment groups are indicated by colored points and ellipses (50% CI). The percentage of the variance explained by each PC is shown in parentheses.

**Additional file 8: Figure S5.** Features with Significant Trait by Treatment Interactions. A heatmap of feature-wise Z-scores among samples by trait (sensitive; resistant) and treatment (Ctr = control; H2O2 = H_2_O_2_) for the 105 features with significant trait-by-treatment interaction (FDR < 0.1). Data are clustered by row.

**Additional file 9: Figure S6.** Glycogen Pathways (a) biosynthesis of glycogen from glucose in metazoans. (b) glycogen degradation into glucose. Data are from BioCyc database (Caspi et al., 2016). Metabolites are in roman font, polypeptides are in bold and biochemical pathways are in italic.

**Additional file 10: Figure S7.** Genes Affecting H_2_O_2_ Resistance may act in Common Pathways Several candidate genes, pathways or processes identified in this study (boxed) are known to regulate feeding and/or glycogen metabolism in response to cues from the nutrient environment. This figure was generated using information from the literature and we attempt to draw parsimonious connections without including many of the possible intermediate genes or signaling events involved. Genes or arrows shown in grey are used to depict hypothetical connections between these pathways or processes.

## Data Availability

All data and R code are available on GitHub: https://github.com/ben6uw/Harrison-et-al-2020-Data-Repository

## Abbreviations

H_2_O_2_: Hydrogen peroxide
GWAS: Genome-wide association studies
DGRP: *Drosophila* genetic reference panel
IlS: Insulin-like signaling
MAF: Minor allele frequency
FDR: False discovery rate
LD: Linkage disequilibrium
SNP: Single nucleotide polymorphism
GO: Gene ontology
RNAi: RNA interference
PCA: Principal component analysis
PC: Principal component
LC-MS: Liquid chromatography mass spectrometry

## References

[1] Boyle EA, Li YI, Pritchard JK (2017). An expanded view of complex traits: from polygenic to Omnigenic. Cell.

[2] Patti GJ, Yanes O, Siuzdak G (2012). Innovation: metabolomics: the apogee of the omics trilogy. Nat Rev Mol Cell Biol.

[3] Johnson CH, Ivanisevic J, Siuzdak G (2016). Metabolomics: beyond biomarkers and towards mechanisms. Nat Rev Mol Cell Biol.

[4] Zampieri M, Sauer U (2017). Metabolomics-driven understanding of genotype-phenotype relations in model organisms. Current Opinion in Systems Biology.

[5] Handakumbura PP, Stanfill B, Rivas-Ubach A, Fortin D, Vogel JP, Jansson C (2019). Metabotyping as a stopover in genome-to-Phenome mapping. Sci Rep.

[6] Fu J, Keurentjes JJ, Bouwmeester H, America T, Verstappen FW, Ward JL, Beale MH, de Vos RC, Dijkstra M, Scheltema RA (2009). System-wide molecular evidence for phenotypic buffering in Arabidopsis. Nat Genet.

[7] Parker BL, Calkin AC, Seldin MM, Keating MF, Tarling EJ, Yang P, Moody SC, Liu Y, Zerenturk EJ, Needham EJ (2019). An integrative systems genetic analysis of mammalian lipid metabolism. Nature.

[8] Wang Z, Klipfell E, Bennett BJ, Koeth R, Levison BS, Dugar B, Feldstein AE, Britt EB, Fu X, Chung YM (2011). Gut flora metabolism of phosphatidylcholine promotes cardiovascular disease. Nature.

[9] Hoffman JM, Soltow QA, Li S, Sidik A, Jones DP, Promislow DE (2014). Effects of age, sex, and genotype on high-sensitivity metabolomic profiles in the fruit fly, Drosophila melanogaster. Aging Cell.

[10] Mahieu NG, Patti GJ (2017). Systems-level annotation of a metabolomics data set reduces 25000 features to fewer than 1000 unique metabolites. Anal Chem.

[11] Fuhrer T, Zamboni N (2015). High-throughput discovery metabolomics. Curr Opin Biotechnol.

[12] Sevin DC, Sauer U (2014). Ubiquinone accumulation improves osmotic-stress tolerance in Escherichia coli. Nat Chem Biol.

[13] Cirulli ET, Guo L, Leon Swisher C, Shah N, Huang L, Napier LA, Kirkness EF, Spector TD, Caskey CT, Thorens B (2019). Profound Perturbation of the Metabolome in Obesity Is Associated with Health Risk. Cell Metab.

[14] Williams S, Dew-Budd K, Davis K, Anderson J, Bishop R, Freeman K, Davis D, Bray K, Perkins L, Hubickey J, Reed LK (2015). Metabolomic and Gene Expression Profiles Exhibit Modular Genetic and Dietary Structure Linking Metabolic Syndrome Phenotypes in Drosophila. G3 (Bethesda).

[15] Shin SY, Fauman EB, Petersen AK, Krumsiek J, Santos R, Huang J, Arnold M, Erte I, Forgetta V, Yang TP (2014). An atlas of genetic influences on human blood metabolites. Nat Genet.

[16] Adamski J, Suhre K (2013). Metabolomics platforms for genome wide association studies--linking the genome to the metabolome. Curr Opin Biotechnol.

[17] Wen W, Li K, Alseekh S, Omranian N, Zhao L, Zhou Y, Xiao Y, Jin M, Yang N, Liu H (2015). Genetic determinants of the network of primary metabolism and their relationships to plant performance in a maize recombinant inbred line population. Plant Cell.

[18] Suhre K, Shin SY, Petersen AK, Mohney RP, Meredith D, Wagele B, Altmaier E, Deloukas P, Erdmann J, Grundberg E (2011). Human metabolic individuality in biomedical and pharmaceutical research. Nature.

[19] Wu S, Tohge T, Cuadros-Inostroza A, Tong H, Tenenboim H, Kooke R, Meret M, Keurentjes JB, Nikoloski Z, Fernie AR (2018). Mapping the Arabidopsis metabolic landscape by untargeted metabolomics at different environmental conditions. Mol Plant.

[20] Klarin D, Damrauer SM, Cho K, Sun YV, Teslovich TM, Honerlaw J, Gagnon DR, DuVall SL, Li J, Peloso GM (2018). Genetics of blood lipids among ~300,000 multi-ethnic participants of the million veteran program. Nat Genet.

[21] Rinschen MM, Ivanisevic J, Giera M, Siuzdak G (2019). Identification of bioactive metabolites using activity metabolomics. Nat Rev Mol Cell Biol.

[22] Huang W, Massouras A, Inoue Y, Peiffer J, Ramia M, Tarone AM, Turlapati L, Zichner T, Zhu D, Lyman RF (2014). Natural variation in genome architecture among 205 Drosophila melanogaster genetic reference panel lines. Genome Res.

[23] Linford NJ, Bilgir C, Ro J, Pletcher SD. Measurement of lifespan in *Drosophila melanogaster*. J Vis Exp. 2013;71:50068. 10.3791/50068 PMC3582515 23328955

[24] Pickering AM, Vojtovich L, Tower J, AD KJ (2013). Oxidative stress adaptation with acute, chronic, and repeated stress. Free Radic Biol Med.

[25] Weber AL, Khan GF, Magwire MM, Tabor CL, Mackay TF, Anholt RR (2012). Genome-wide association analysis of oxidative stress resistance in Drosophila melanogaster. PLoS One.

[26] Mackay TF, Richards S, Stone EA, Barbadilla A, Ayroles JF, Zhu D, Casillas S, Han Y, Magwire MM, Cridland JM (2012). The Drosophila melanogaster genetic reference panel. Nature.

[27] Mackay TF (2002). The nature of quantitative genetic variation for Drosophila longevity. Mech Ageing Dev.

[28] Ivanov DK, Escott-Price V, Ziehm M, Magwire MM, Mackay TF, Partridge L, Thornton JM (2015). Longevity GWAS using the Drosophila genetic reference panel. J Gerontol A Biol Sci Med Sci.

[29] Jordan KW, Craver KL, Magwire MM, Cubilla CE, Mackay TF, Anholt RR (2012). Genome-wide association for sensitivity to chronic oxidative stress in Drosophila melanogaster. PLoS One.

[30] King EG, Macdonald SJ, Long AD. Properties and power of the Drosophila synthetic population resource for the routine dissection of complex traits. Genetics. 2012;191:935-49. 10.1534/genetics.112.138537 PMC3389985 22505626

[31] Reed LK, Lee K, Zhang Z, Rashid L, Poe A, Hsieh B, Deighton N, Glassbrook N, Bodmer R, Gibson G (2014). Systems genomics of metabolic phenotypes in wild-type Drosophila melanogaster. Genetics.

[32] Hariharan R, Hoffman JM, Thomas AS, Soltow QA, Jones DP, Promislow DE (2014). Invariance and plasticity in the Drosophila melanogaster metabolomic network in response to temperature. BMC Syst Biol.

[33] Malmendal A, Overgaard J, Bundy JG, Sorensen JG, Nielsen NC, Loeschcke V, Holmstrup M (2006). Metabolomic profiling of heat stress: hardening and recovery of homeostasis in Drosophila. Am J Physiol Regul Integr Comp Physiol.

[34] Overgaard J, Malmendal A, Sorensen JG, Bundy JG, Loeschcke V, Nielsen NC, Holmstrup M (2007). Metabolomic profiling of rapid cold hardening and cold shock in Drosophila melanogaster. J Insect Physiol.

[35] Coquin L, Feala JD, McCulloch AD, Paternostro G (2008). Metabolomic and flux-balance analysis of age-related decline of hypoxia tolerance in Drosophila muscle tissue. Mol Syst Biol.

[36] An PNT, Shimaji K, Tanaka R, Yoshida H, Kimura H, Fukusaki E, Yamaguchi M (2017). Epigenetic regulation of starvation-induced autophagy in Drosophila by histone methyltransferase G9a. Sci Rep.

[37] Everman ER, Morgan TJ (2018). Antagonistic pleiotropy and mutation accumulation contribute to age-related decline in stress response. Evolution.

[38] Ja WW, Carvalho GB, Mak EM, de la Rosa NN, Fang AY, Liong JC, Brummel T, Benzer S (2007). Prandiology of Drosophila and the CAFE assay. Proc Natl Acad Sci U S A.

[39] Wong R, Piper MD, Wertheim B, Partridge L (2009). Quantification of food intake in Drosophila. PLoS One.

[40] Purcell S, Neale B, Todd-Brown K, Thomas L, Ferreira MA, Bender D, Maller J, Sklar P, de Bakker PI, Daly MJ, Sham PC (2007). PLINK: a tool set for whole-genome association and population-based linkage analyses. Am J Hum Genet.

[41] Storey JD, Tibshirani R (2003). Statistical significance for genomewide studies. Proc Natl Acad Sci U S A.

[42] Mirina A, Atzmon G, Ye K, Bergman A (2012). Gene size matters. PLoS One.

[43] Hyun S, Lee JH, Jin H, Nam J, Namkoong B, Lee G, Chung J, Kim VN (2009). Conserved MicroRNA miR-8/miR-200 and its target USH/FOG2 control growth by regulating PI3K. Cell.

[44] Roman G, Endo K, Zong L, Davis RL (2001). P [switch], a system for spatial and temporal control of gene expression in Drosophila melanogaster. Proc Natl Acad Sci U S A.

[45] Fadda M, Hasakiogullari I, Temmerman L, Beets I, Zels S, Schoofs L (2019). Regulation of feeding and metabolism by neuropeptide F and short neuropeptide F in invertebrates. Front Endocrinol (Lausanne).

[46] Li S, Park Y, Duraisingham S, Strobel FH, Khan N, Soltow QA, Jones DP, Pulendran B (2013). Predicting network activity from high throughput metabolomics. PLoS Comput Biol.

[47] Yamada T, Habara O, Kubo H, Nishimura T. Fat body glycogen serves as a metabolic safeguard for the maintenance of sugar levels in Drosophila. Development. 2018;145:dev165910. 10.1242/dev.158865 29467247

[48] Srivastav S, Singh SK, Yadav AK, Srikrishna S (2015). Folic acid supplementation rescues anomalies associated with knockdown of parkin in dopaminergic and serotonergic neurons in Drosophila model of Parkinson's disease. Biochem Biophys Res Commun.

[49] Jansen PR, Watanabe K, Stringer S, Skene N, Bryois J, Hammerschlag AR, de Leeuw CA, Benjamins JS, Munoz-Manchado AB, Nagel M (2019). Genome-wide analysis of insomnia in 1,331,010 individuals identifies new risk loci and functional pathways. Nat Genet.

[50] Timmers PR, Mounier N, Lall K, Fischer K, Ning Z, Feng X, Bretherick AD, Clark DW, Agbessi M, Ahsan H, et al. Genomics of 1 million parent lifespans implicates novel pathways and common diseases and distinguishes survival chances. Elife. 2019;8:e39856. 10.7554/eLife.39856 PMC6333444 30642433

[51] Park JH, Wacholder S, Gail MH, Peters U, Jacobs KB, Chanock SJ, Chatterjee N (2010). Estimation of effect size distribution from genome-wide association studies and implications for future discoveries. Nat Genet.

[52] Zhang Y, Qi G, Park JH, Chatterjee N (2018). Estimation of complex effect-size distributions using summary-level statistics from genome-wide association studies across 32 complex traits. Nat Genet.

[53] Pilling LC, Kuo CL, Sicinski K, Tamosauskaite J, Kuchel GA, Harries LW, Herd P, Wallace R, Ferrucci L, Melzer D (2017). Human longevity: 25 genetic loci associated in 389,166 UK biobank participants. Aging (Albany NY).

[54] Yang J, Zeng J, Goddard ME, Wray NR, Visscher PM (2017). Concepts, estimation and interpretation of SNP-based heritability. Nat Genet.

[55] Mackay TFC, Huang W. Charting the genotype-phenotype map: lessons from the Drosophila melanogaster genetic reference panel. Wiley Interdiscip Rev Dev Biol. 2018;7:10.1002/wdev.289. 10.1002/wdev.289 PMC5746472 28834395

[56] Ober U, Ayroles JF, Stone EA, Richards S, Zhu D, Gibbs RA, Stricker C, Gianola D, Schlather M, Mackay TF, Simianer H (2012). Using whole-genome sequence data to predict quantitative trait phenotypes in Drosophila melanogaster. PLoS Genet.

[57] Huang W, Richards S, Carbone MA, Zhu D, Anholt RR, Ayroles JF, Duncan L, Jordan KW, Lawrence F, Magwire MM (2012). Epistasis dominates the genetic architecture of Drosophila quantitative traits. Proc Natl Acad Sci U S A.

[58] Fiehn O (2002). Metabolomics--the link between genotypes and phenotypes. Plant Mol Biol.

[59] Avanesov AS, Ma S, Pierce KA, Yim SH, Lee BC, Clish CB, Gladyshev VN (2014). Age- and diet-associated metabolome remodeling characterizes the aging process driven by damage accumulation. Elife.

[60] Laye MJ, Tran V, Jones DP, Kapahi P, Promislow DE (2015). The effects of age and dietary restriction on the tissue-specific metabolome of Drosophila. Aging Cell.

[61] MacMillan HA, Knee JM, Dennis AB, Udaka H, Marshall KE, Merritt TJ, Sinclair BJ (2016). Cold acclimation wholly reorganizes the Drosophila melanogaster transcriptome and metabolome. Sci Rep.

[62] Knee JM, Rzezniczak TZ, Barsch A, Guo KZ, Merritt TJ (2013). A novel ion pairing LC/MS metabolomics protocol for study of a variety of biologically relevant polar metabolites. J Chromatogr B Analyt Technol Biomed Life Sci.

[63] Doran ML, Knee JM, Wang N, Rzezniczak TZ, Parkes TL, Li L, Merritt TJS. Metabolomic analysis of oxidative stress: superoxide dismutase mutation and paraquat induced stress in Drosophila melanogaster. Free Radic Biol Med. 2017;113:323–34. 10.1016/j.freeradbiomed.2017.10.011 29031835

[64] Zampieri M, Zimmermann M, Claassen M, Sauer U (2017). Nontargeted metabolomics reveals the multilevel response to antibiotic perturbations. Cell Rep.

[65] Shukla AK, Ratnasekhar C, Pragya P, Chaouhan HS, Patel DK, Chowdhuri DK, Mudiam MKR (2016). Metabolomic analysis provides insights on Paraquat-induced Parkinson-like symptoms in Drosophila melanogaster. Mol Neurobiol.

[66] Rzezniczak TZ, Merritt TJ (2012). Interactions of NADP-reducing enzymes across varying environmental conditions: a model of biological complexity. G3 (Bethesda).

[67] Riahi H, Brekelmans C, Foriel S, Merkling SH, Lyons TA, Itskov PM, Kleefstra T, Ribeiro C, van Rij RP, Kramer JM, Schenck A (2019). The histone methyltransferase G9a regulates tolerance to oxidative stress-induced energy consumption. PLoS Biol.

[68] Hwangbo DS, Gershman B, Tu MP, Palmer M, Tatar M (2004). Drosophila dFOXO controls lifespan and regulates insulin signalling in brain and fat body. Nature.

[69] Poirier L, Shane A, Zheng J, Seroude L (2008). Characterization of the Drosophila gene-switch system in aging studies: a cautionary tale. Aging Cell.

[70] Bauer M, Katzenberger JD, Hamm AC, Bonaus M, Zinke I, Jaekel J, Pankratz MJ (2006). Purine and folate metabolism as a potential target of sex-specific nutrient allocation in Drosophila and its implication for lifespan-reproduction tradeoff. Physiol Genomics.

[71] Yu S, Jang Y, Paik D, Lee E, Park JJ (2015). Nmdmc overexpression extends Drosophila lifespan and reduces levels of mitochondrial reactive oxygen species. Biochem Biophys Res Commun.

[72] Celardo I, Lehmann S, Costa AC, Loh SH, Miguel Martins L (2017). dATF4 regulation of mitochondrial folate-mediated one-carbon metabolism is neuroprotective. Cell Death Differ.

[73] Landis GN, Abdueva D, Skvortsov D, Yang J, Rabin BE, Carrick J, Tavaré S, Tower J (2004). Similar gene expression patterns characterize aging and oxidative stress in Drosophila melanogaster. Proc Natl Acad Sci U S A.

[74] Girardot F, Monnier V, Tricoire H (2004). Genome wide analysis of common and specific stress responses in adult drosophila melanogaster. BMC Genomics.

[75] Tufi R, Gandhi S, de Castro IP, Lehmann S, Angelova PR, Dinsdale D, Deas E, Plun-Favreau H, Nicotera P, Abramov AY (2014). Enhancing nucleotide metabolism protects against mitochondrial dysfunction and neurodegeneration in a PINK1 model of Parkinson's disease. Nat Cell Biol.

[76] Curtis C, Landis GN, Folk D, Wehr NB, Hoe N, Waskar M, Abdueva D, Skvortsov D, Ford D, Luu A (2007). Transcriptional profiling of MnSOD-mediated lifespan extension in Drosophila reveals a species-general network of aging and metabolic genes. Genome Biol.

[77] Landis G, Shen J, Tower J (2012). Gene expression changes in response to aging compared to heat stress, oxidative stress and ionizing radiation in *Drosophila melanogaster*. Aging (Albany NY).

[78] Colinet H, Renault D (2018). Similar post-stress metabolic trajectories in young and old flies. Exp Gerontol.

[79] Ding MZ, Li BZ, Cheng JS, Yuan YJ (2010). Metabolome analysis of differential responses of diploid and haploid yeast to ethanol stress. Omics.

[80] Nikiforova VJ, Kopka J, Tolstikov V, Fiehn O, Hopkins L, Hawkesford MJ, Hesse H, Hoefgen R (2005). Systems rebalancing of metabolism in response to sulfur deprivation, as revealed by metabolome analysis of Arabidopsis plants. Plant Physiol.

[81] Shorter J, Couch C, Huang W, Carbone MA, Peiffer J, Anholt RR, Mackay TF (2015). Genetic architecture of natural variation in Drosophila melanogaster aggressive behavior. Proc Natl Acad Sci U S A.

[82] Enattah NS, Sahi T, Savilahti E, Terwilliger JD, Peltonen L, Jarvela I (2002). Identification of a variant associated with adult-type hypolactasia. Nat Genet.

[83] Wu Q, Zhao Z, Shen P (2005). Regulation of aversion to noxious food by Drosophila neuropeptide Y- and insulin-like systems. Nat Neurosci.

[84] Krashes MJ, DasGupta S, Vreede A, White B, Armstrong JD, Waddell S (2009). A neural circuit mechanism integrating motivational state with memory expression in Drosophila. Cell.

[85] Britton JS, Lockwood WK, Li L, Cohen SM, Edgar BA (2002). Drosophila's insulin/PI3-kinase pathway coordinates cellular metabolism with nutritional conditions. Dev Cell.

[86] Junger MA, Rintelen F, Stocker H, Wasserman JD, Vegh M, Radimerski T, Greenberg ME, Hafen E (2003). The Drosophila forkhead transcription factor FOXO mediates the reduction in cell number associated with reduced insulin signaling. J Biol.

[87] Tettweiler G, Miron M, Jenkins M, Sonenberg N, Lasko PF (2005). Starvation and oxidative stress resistance in Drosophila are mediated through the eIF4E-binding protein, d4E-BP. Genes Dev.

[88] Teleman AA (2009). Molecular mechanisms of metabolic regulation by insulin in Drosophila. Biochem J.

[89] Bjedov I, Toivonen JM, Kerr F, Slack C, Jacobson J, Foley A, Partridge L (2010). Mechanisms of life span extension by rapamycin in the fruit fly Drosophila melanogaster. Cell Metab.

[90] Broughton SJ, Piper MDW, Ikeya T, Bass TM, Jacobson J, Driege Y, Martinez P, Hafen E, Withers DJ, Leevers SJ, Partridge L (2005). Longer lifespan, altered metabolism, and stress resistance in <em>Drosophila</em> from ablation of cells making insulin-like ligands. Proc Natl Acad Sci U S A.

[91] Buch S, Melcher C, Bauer M, Katzenberger J, Pankratz MJ (2008). Opposing effects of dietary protein and sugar regulate a transcriptional target of Drosophila insulin-like peptide signaling. Cell Metab.

[92] Ainsley JA, Kim MJ, Wegman LJ, Pettus JM, Johnson WA (2008). Sensory mechanisms controlling the timing of larval developmental and behavioral transitions require the Drosophila DEG/ENaC subunit, Pickpocket1. Dev Biol.

[93] Freeman EG, Dahanukar A (2015). Molecular neurobiology of Drosophila taste. Curr Opin Neurobiol.

[94] Olds WH, Xu T. Regulation of food intake by mechanosensory ion channels in enteric neurons. Elife. 2014;3:e04402. 10.7554/eLife.04402 PMC4225495 25285450

[95] Wegman LJ, Ainsley JA, Johnson WA (2010). Developmental timing of a sensory-mediated larval surfacing behavior correlates with cessation of feeding and determination of final adult size. Dev Biol.

[96] Waterson MJ, Chung BY, Harvanek ZM, Ostojic I, Alcedo J, Pletcher SD (2014). Water sensor ppk28 modulates Drosophila lifespan and physiology through AKH signaling. Proc Natl Acad Sci U S A.

[97] Branch A, Shen P: Central and Peripheral Regulation of Appetite and Food Intake in Drosophila. In Appetite and Food Intake: Central Control*.* Edited by nd, Harris RBS. Boca Raton: CRC Press/Taylor & Francis (c) 2017 By Taylor & Francis Group, LLC. 2017: 17–38. 28880507

[98] Vigne P, Frelin C (2010). Hypoxia modifies the feeding preferences of Drosophila. Consequences for diet dependent hypoxic survival. BMC Physiol.

[99] Edgecomb RS, Harth CE, Schneiderman AM (1994). Regulation of feeding behavior in adult Drosophila melanogaster varies with feeding regime and nutritional state. J Exp Biol.

[100] Min KJ, Tatar M (2006). Drosophila diet restriction in practice: do flies consume fewer nutrients?. Mech Ageing Dev.

[101] Deshpande SA, Yamada R, Mak CM, Hunter B, Soto Obando A, Hoxha S, Ja WW (2015). Acidic food pH increases palatability and consumption and extends Drosophila lifespan. J Nutr.

[102] Zeng C, Du Y, Alberico T, Seeberger J, Sun X, Zou S (2011). Gender-specific prandial response to dietary restriction and oxidative stress in Drosophila melanogaster. Fly (Austin).

[103] Galikova M, Diesner M, Klepsatel P, Hehlert P, Xu Y, Bickmeyer I, Predel R, Kuhnlein RP (2015). Energy homeostasis control in Drosophila Adipokinetic hormone mutants. Genetics.

[104] Kim MJ, Ainsley JA, Carder JW, Johnson WA (2013). Hyperoxia-triggered aversion behavior in Drosophila foraging larvae is mediated by sensory detection of hydrogen peroxide. J Neurogenet.

[105] Guntur AR, Gou B, Gu P, He R, Stern U, Xiang Y, Yang CH (2017). H2O2-sensitive isoforms of Drosophila melanogaster TRPA1 act in bitter-sensing gustatory neurons to promote avoidance of UV during egg-laying. Genetics.

[106] Bhatla N, Horvitz HR (2015). Light and hydrogen peroxide inhibit C. elegans feeding through gustatory receptor orthologs and pharyngeal neurons. Neuron.

[107] Ameku T, Yoshinari Y, Texada MJ, Kondo S, Amezawa K, Yoshizaki G, Shimada-Niwa Y, Niwa R (2018). Midgut-derived neuropeptide F controls germline stem cell proliferation in a mating-dependent manner. PLoS Biol.

[108] R Core Team (2018). R: a language and environment for statistical computing. Vienna, Austria: R Foundation for Statistical Computing.

[109] Terry T (2015). A Package for Survival Analysis in S . version 2.38.

[110] Garlapow ME, Huang W, Yarboro MT, Peterson KR, Mackay TF (2015). Quantitative genetics of food intake in Drosophila melanogaster. PLoS One.

[111] Xavier A, Xu S, Muir WM, Rainey KM (2015). NAM: association studies in multiple populations. Bioinformatics.

[112] Yang J, Lee SH, Goddard ME, Visscher PM (2011). GCTA: a tool for genome-wide complex trait analysis. Am J Hum Genet.

[113] Patterson N, Price AL, Reich D (2006). Population structure and eigenanalysis. PLoS Genet.

[114] Gramates LS, Marygold SJ, Santos GD, Urbano JM, Antonazzo G, Matthews BB, Rey AJ, Tabone CJ, Crosby MA, Emmert DB (2017). FlyBase at 25: looking to the future. Nucleic Acids Res.

[115] Mi HHXMATHMCKDTPD (2017). PANTHER version 11: expanded annotation data from gene ontology and Reactome pathways, and data analysis tool enhancements. Nucleic Acids Res.

[116] Members MSIB, Sansone SA, Fan T, Goodacre R, Griffin JL, Hardy NW, Kaddurah-Daouk R, Kristal BS, Lindon J, Mendes P (2007). The metabolomics standards initiative. Nat Biotechnol.

[117] Troyanskaya O, Cantor M, Sherlock G, Brown P, Hastie T, Tibshirani R, Botstein D, Altman RB (2001). Missing value estimation methods for DNA microarrays. Bioinformatics.

[118] Ritchie ME, Phipson B, Wu D, Hu Y, Law CW, Shi W, Smyth GK (2015). limma powers differential expression analyses for RNA-sequencing and microarray studies. Nucleic Acids Res.

[119] Benjamini Y, Hochberg Y (1995). Controlling the false discovery rate: a practical and powerful approach to multiple testing. J R Stat Soc Ser B Methodol.

