# Supplementary figures and images for: The metabolome as a link in the genotype-phenotype map for peroxide resistance in the fruit fly, Drosophila melanogaster
Source: BMC Genomics. 2020 May 4;21:341. doi: 10.1186/s12864-020-6739-1 (PMC7199327; doi:10.1186/s12864-020-6739-1)

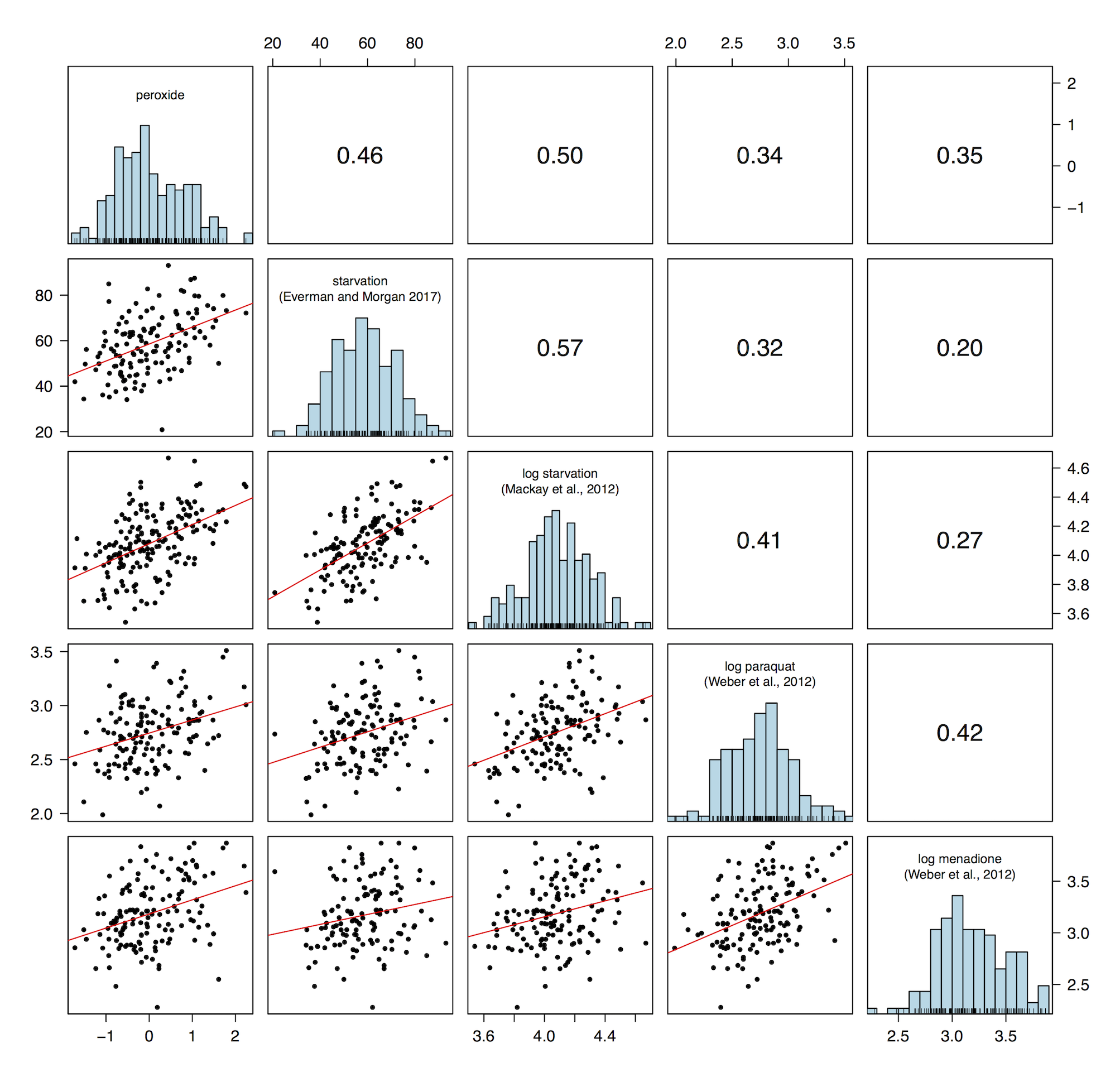

Supplement: Supplementary file 4 — Additional file 4: Figure S1. Trait Correlation Analysis within the DGRP. The correlations of peroxide lifespan (scaled) with several other traits are shown. The traits include lifespan during exposure to starvation [26, 37], or to the log-transformed lifespans during exposure to oxidative stressors paraquat or menadione bisulfite (menadione) [25]. Least-squares regressions are shown in red. Above the diagonal are Pearson’s correlation coefficients for each pair of traits. There is significant correlation for peroxide survival with each of the traits shown (P < 0.05, corrected for multiple comparisons). [file 12864_2020_6739_MOESM4_ESM.tif]

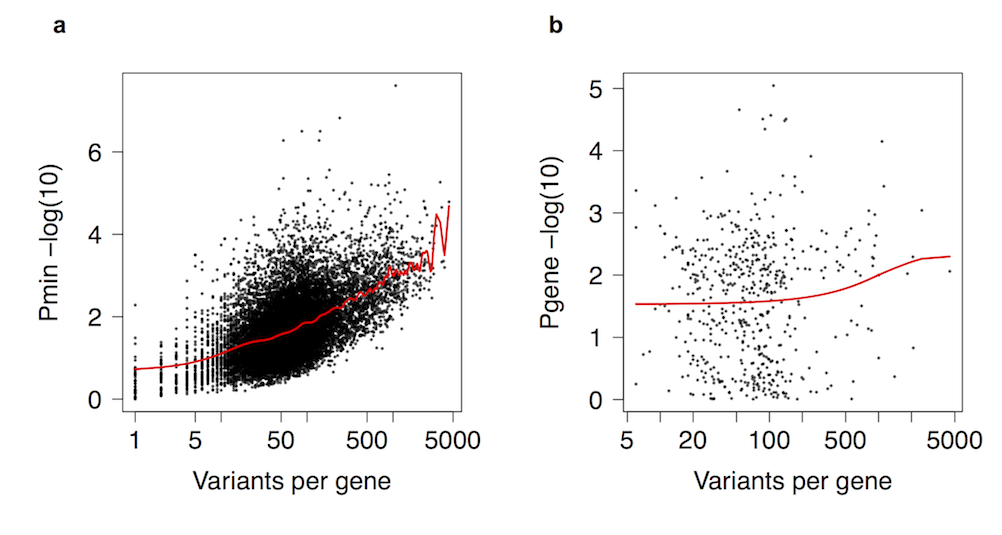

Supplement: Supplementary file 5 — Additional file 5: Figure S2. Permutation Approach Corrects for Bias in Gene-Level Associations (a) –log10 Pmin for 4810 gene models plotted over the number of variants per gene. (b) –log10 Pgene for 533 gene models that were permuted 1 million times plotted over the number of variants per gene. The red lines in (a) and (b) are cubic splines using the default parameters in the R function smoothspline. [file 12864_2020_6739_MOESM5_ESM.tif]

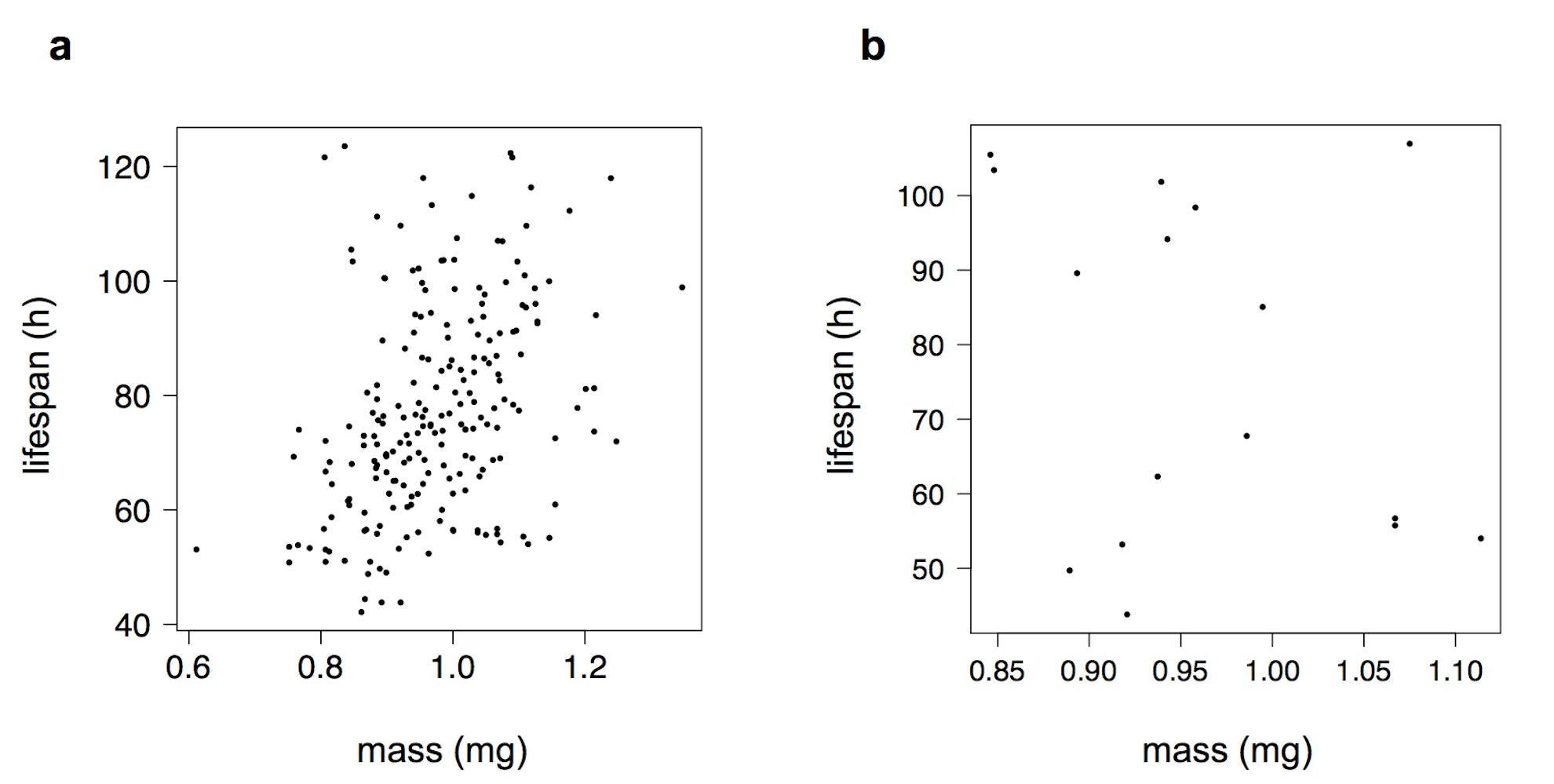

Supplement: Supplementary file 6 — Additional file 6: Figure S3. Lifespan and Fly Mass and the Selection of Lines for Metabolomics Mean lifespan (n = 17 to 80 flies) for each DGRP line over the average fly mass (n = 5 to 25 flies). (a) Linear modeling found a significant interaction between mean lifespan from all blocks and fly mass (P = 1.6 × 10− 5). Mass remained significant across the study after correcting for block effects (P = 7.5 × 10− 6). (b) The lifespans of lines chosen for metabolomics minimized the effect of mass on lifespan (P = 0.171). [file 12864_2020_6739_MOESM6_ESM.tif]

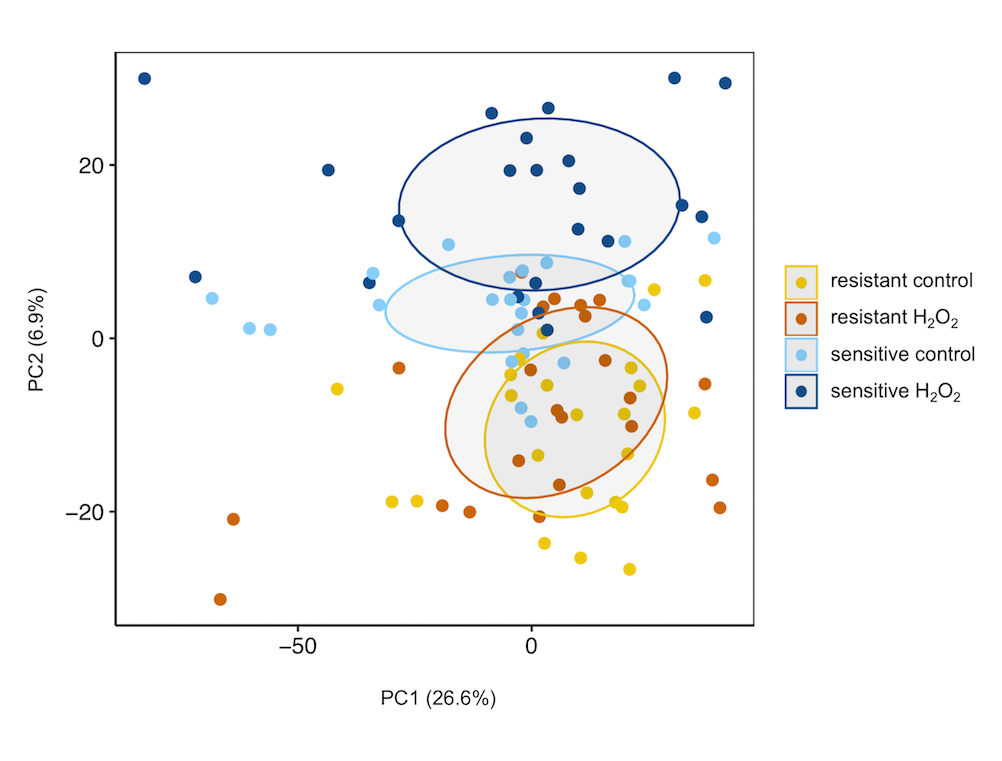

Supplement: Supplementary file 7 — Additional file 7: Figure S4. Projections of Metabolomic Principal Components The first and second principal components of the positive mode data labeled to indicate resistant and sensitive genotypes, as well as the effect of treatment (control vs. H2O2). Trait and treatment groups are indicated by colored points and ellipses (50% CI). The percentage of the variance explained by each PC is shown in parentheses. [file 12864_2020_6739_MOESM7_ESM.tif]

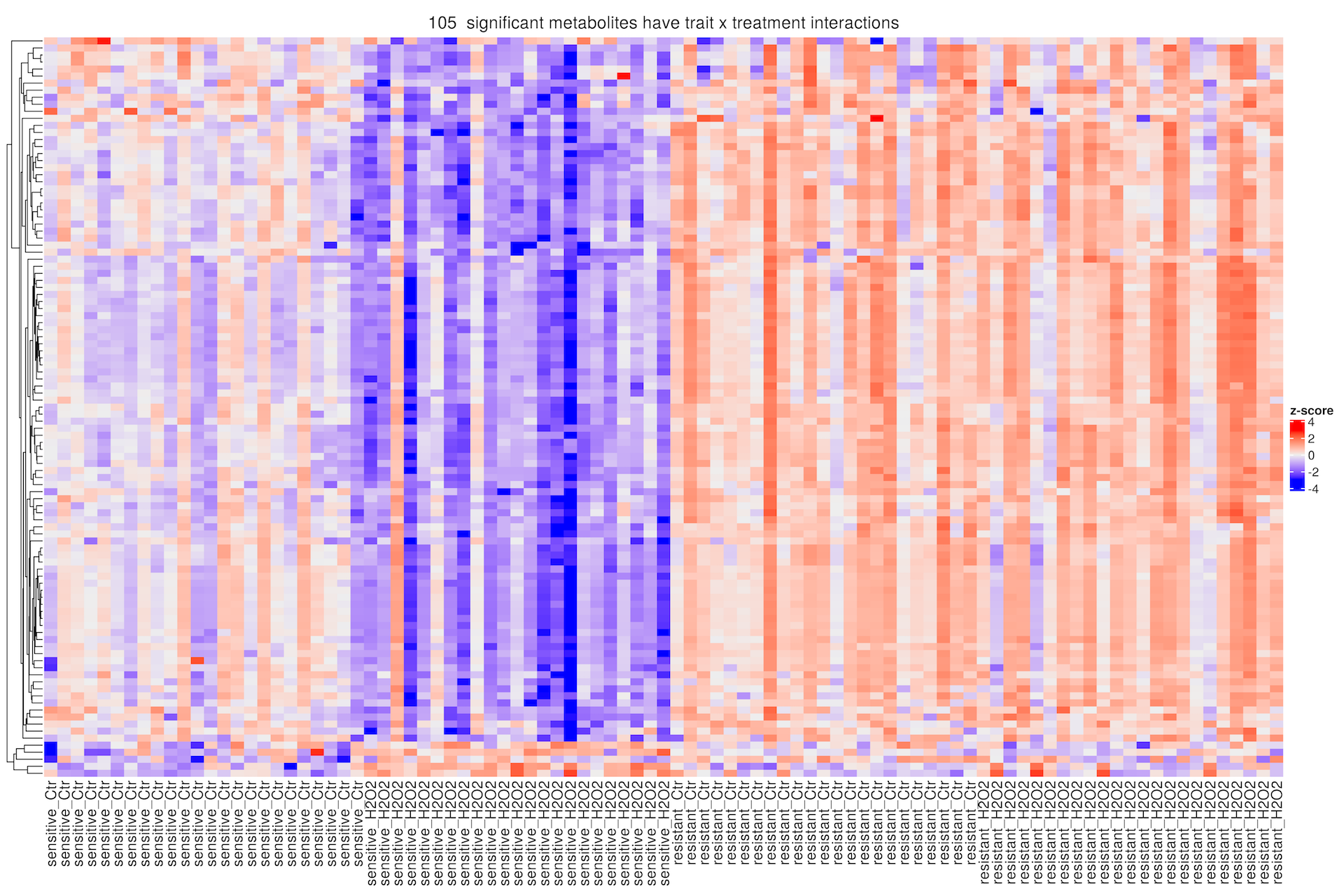

Supplement: Supplementary file 8 — Additional file 8: Figure S5. Features with Significant Trait by Treatment Interactions. A heatmap of feature-wise Z-scores among samples by trait (sensitive; resistant) and treatment (Ctr = control; H2O2 = H2O2) for the 105 features with significant trait-by-treatment interaction (FDR < 0.1). Data are clustered by row. [file 12864_2020_6739_MOESM8_ESM.tif]

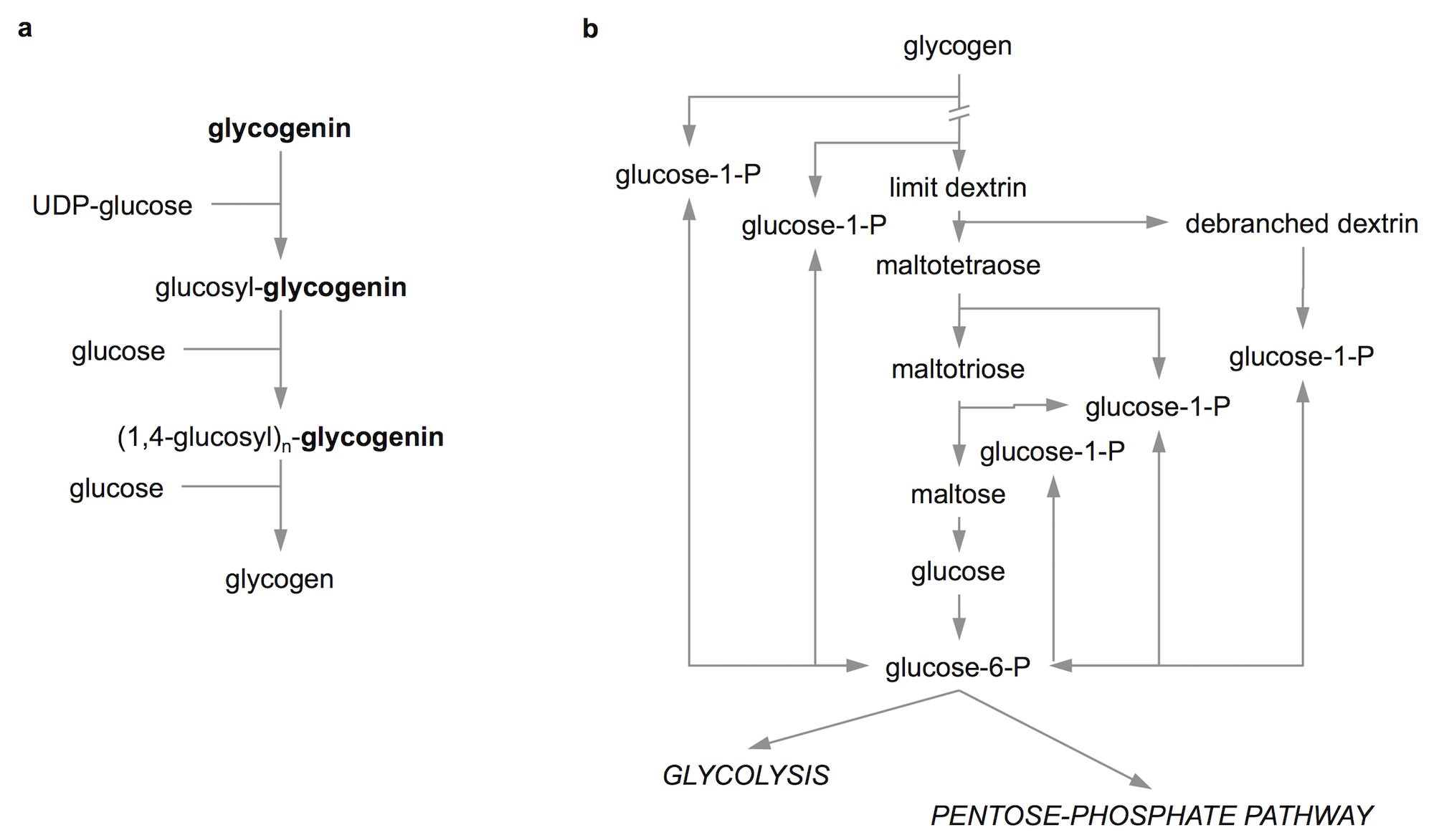

Supplement: Supplementary file 9 — Additional file 9: Figure S6. Glycogen Pathways (a) biosynthesis of glycogen from glucose in metazoans. (b) glycogen degradation into glucose. Data are from BioCyc database (Caspi et al., 2016). Metabolites are in roman font, polypeptides are in bold and biochemical pathways are in italic. [file 12864_2020_6739_MOESM9_ESM.tif]

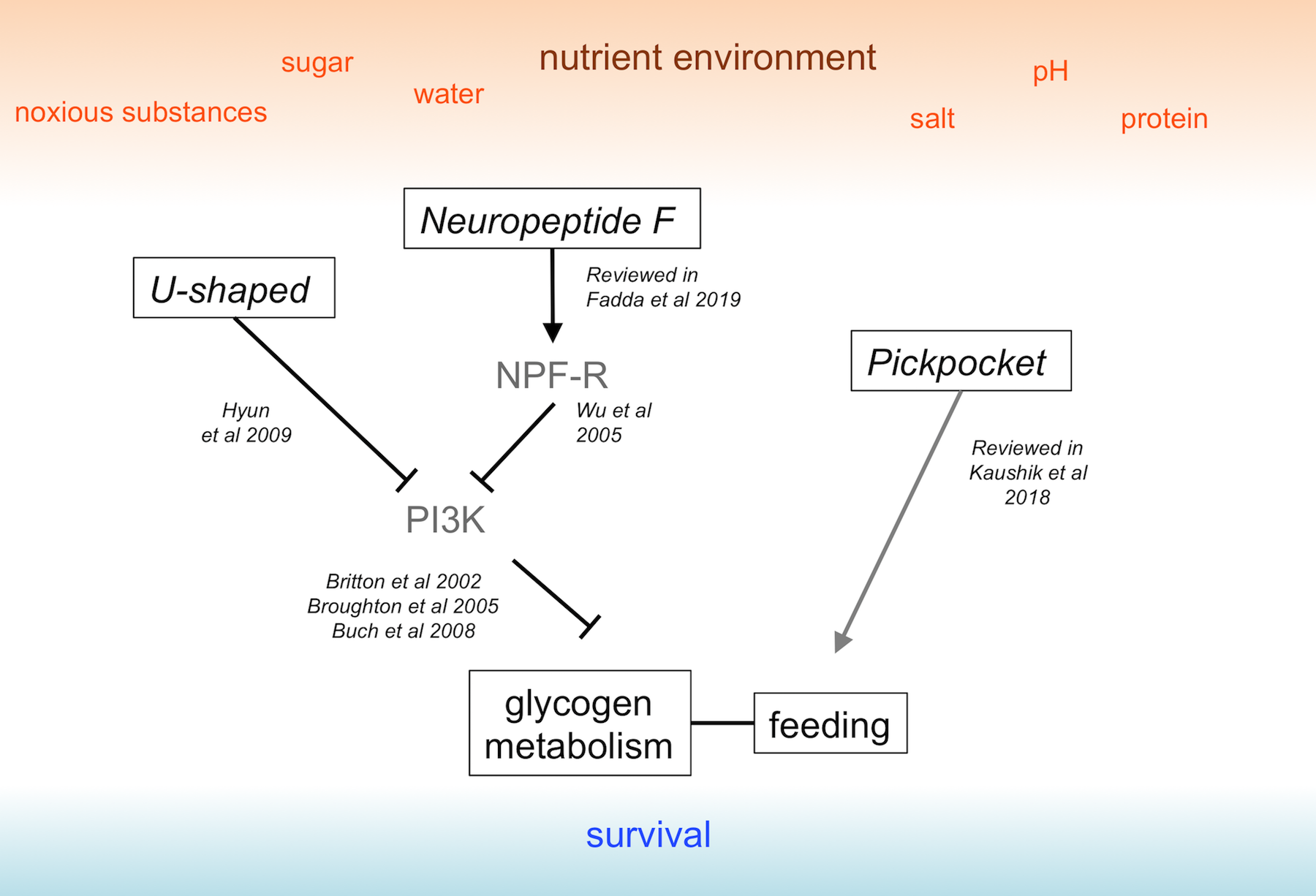

Supplement: Supplementary file 10 — Additional file 10: Figure S7. Genes Affecting H2O2 Resistance may act in Common Pathways Several candidate genes, pathways or processes identified in this study (boxed) are known to regulate feeding and/or glycogen metabolism in response to cues from the nutrient environment. This figure was generated using information from the literature and we attempt to draw parsimonious connections without including many of the possible intermediate genes or signaling events involved. Genes or arrows shown in grey are used to depict hypothetical connections between these pathways or processes. [file 12864_2020_6739_MOESM10_ESM.tif]
